# Differential Effects of β-catenin and NF-κB Interplay in the Regulation of Cell Proliferation, Inflammation and Tumorigenesis in Response to Bacterial Infection

**DOI:** 10.1371/journal.pone.0079432

**Published:** 2013-11-21

**Authors:** Parthasarathy Chandrakesan, Laxmi Uma Maheswar Rao Jakkula, Ishfaq Ahmed, Badal Roy, Shrikant Anant, Shahid Umar

**Affiliations:** 1 Department of Internal Medicine, Division of Digestive Diseases and Nutrition, University of Oklahoma Health Sciences Center, Oklahoma City, Oklahoma, United States of America; 2 Department of Molecular and Integrative Physiology, University of Kansas Medical Center, Kansas City, Kansas, United States of America; University of Kentucky, United States of America

## Abstract

Both β-catenin and NF-κB have been implicated in our laboratory as candidate factors in driving proliferation in an *in vivo* model of *Citrobacter rodentium* (CR)-induced colonic crypt hyper-proliferation and hyperplasia. Herein, we test the hypothesis that β-catenin and not necessarily NF-κB regulates colonic crypt hyperplasia or tumorigenesis in response to CR infection. When C57Bl/6 wild type (WT) mice were infected with CR, sequential increases in proliferation at days 9 and 12 plateaued off at day 19 and paralleled increases in NF-κB signaling. In *Tlr4^−/−^* (KO) mice, a sequential but sustained proliferation which tapered off only marginally at day 19, was associated with TLR4-dependent and independent increases in NF-κB signaling. Similarly, increases in either activated or total β-catenin in the colonic crypts of WT mice as early as day 3 post-infection coincided with cyclinD1 and c-myc expression and associated crypt hyperplasia. In KO mice, a delayed kinetics associated predominantly with increases in non-phosphorylated (active) β-catenin coincided with increases in cyclinD1, c-myc and crypt hyperplasia. Interestingly, PKCζ-catalyzed Ser-9 phosphorylation and inactivation of GSK-3β and not loss of wild type APC protein accounted for β-catenin accumulation and nuclear translocation in either strain. *In vitro* studies with Wnt2b and Wnt5a further validated the interplay between the Wnt/β-catenin and NF-κB pathways, respectively. When WT or KO mice were treated with nanoparticle-encapsulated siRNA to β-catenin (si- β-Cat), almost complete loss of nuclear β-catenin coincided with concomitant decreases in CD44 and crypt hyperplasia without defects in NF-κB signaling. si-β-Cat treatment to *Apc* ^Min/+^ mice attenuated CR-induced increases in β-catenin and CD44 that halted the growth of mutated crypts without affecting NF-κB signaling. The predominant β-catenin-induced crypt proliferation was further validated in a Castaneus strain (B6.CAST.11M) that exhibited significant crypt hyperplasia despite an attenuated NF-κB signaling. Thus, β-catenin and not necessarily NF-κB regulates crypt hyperplasia in response to bacterial infection.

## Introduction

Both β-catenin and NF-κB proteins are important regulators of gene expression and cellular proliferation. β-catenin is a ubiquitously expressed protein that acts as an adhesion molecule by mediating the association of E-cadherin with the cytoskeleton [1], [2] and is also a critical downstream component of the Wnt pathway [3]–[5]. The Wnt family of secretory glycoproteins plays an important role in embryonic development, in the induction of cell polarity, and in the determination of cell fate. Most mammalian genomes including human, harbor 19 Wnt genes, falling into 12 conserved Wnt subfamilies. Wnt proteins are ∼40 kDa in size and contain many conserved cysteines [6]. Wnt ligands produce their effects through activation of one of a dozen different signaling pathways [7]. These pathways are broadly categorized as canonical if they require signaling via β-catenin while non-canonical Wnt signaling pathways are β-catenin independent. Interestingly, multiple studies have demonstrated a regulatory cross-talk between canonical and non-canonical pathways wherein, Wnt5a has been shown to induce a non-canonical pathway that inhibits canonical Wnt signaling. This observation has been made both *in vivo* and *in vitro*, including hematopoietic stem cells [8], [9]. The outcome however, depends on the receptor context [10]. Wnt5a inhibits β-catenin-dependent activity when its signal is mediated by the orphan tyrosine kinase receptor Ror2 [11]. However, it activates β-catenin-dependent signaling when it signals through the co-receptors Fzd4 and LRP5.

NF-κB comprises a family of transcription factors which are critical in activating the expression of genes involved in the immune and inflammatory response and in the regulation of cellular apoptosis [12], [13]. Typically, NF-κB is activated by inflammatory mediators such as TNF-α and IL-1β, via activation of IκB kinases (IKKs), which carry out the phosphorylation-dependent degradation of IκB inhibitors upon inflammatory stimuli [14]–[18]. Constitutively active NF-κB plays an important role in cancer progression and metastasis and has been reported to physically interact with β-catenin, resulting in a reduction in NF-κB nucleus translocation, DNA binding and target gene expression. Recently, GSK-3β is shown to be required for NF-κB activation [19]. As β-catenin is a major substrate of GSK-3β, it raises an interesting possibility that β-catenin may mediate the cross-regulation between the two pathways. Interestingly, β-catenin-independent Wnt signaling is also involved in various biological functions, such as vertebral development, cell motility and adhesion, and cancer invasiveness [20]–[23]. Indeed, Wnt5a has been recently implicated in certain inflammatory diseases [24], [25]. Wnt5a expression is dependent on NF-κB signaling via Toll-like receptor (TLR) in human antigen-presenting cells [25], [26]. Furthermore, IL-6 family members activate the gp130-JAK-signal transducer and activator of transcription 3 (STAT3) signaling cascade to up-regulate Wnt5a transcription in chronic persistent inflammation and rheumatoid arthritis [27]. However, little is known about the expression and modulation of non-canonical Wnt signaling in the intestine in response to bacterial infection.

It has been shown previously that development of necrotizing enterocolitis (NEC), a leading cause of death from gastrointestinal disease in preterm infants, requires activation of TLR4 on enterocytes and that TLR4 activation by bacterial endotoxin LPS (lipopolysaccharide) inhibits enterocyte proliferation and leads to disease progression due to impaired β-catenin signaling after activation of the Akt-GSK3β signaling pathway [28]–[30]. These effects were however, restricted to the small intestine of newborn mice, suggesting relevance in the development of NEC. Whether TLR4-mediated inhibition of β-catenin signaling in response to infection by Gram negative bacteria is a global phenomenon is not known.

*Citrobacter rodentium* (CR) naturally infects mice using a mechanism similar to those employed by attaching and effacing (A/E) bacterial pathogens EPEC and EHEC [31], [32]. CR is an A/E pathogen which causes increased proliferation in the distal colon of adult outbred mice without associated injury or significant histological inflammation [33]. In genetically susceptible strains, clinical signs such as retarded growth, diarrhea, dehydration, coat ruffling, hunched pasture and high mortality have been reported [33]. Utilizing the CR-infection model, we showed for the first time that colonic crypt hyperplasia was associated with NF-κB activation [34] and alterations in Casein Kinase-Iε that influenced β-catenin signaling [35], [36]. Ongoing studies from our laboratory have further demonstrated that functional cross-talk between Wnt/β-catenin and Notch [37] and Notch and NF-κB [38] pathways regulate crypt hyperplasia and/or tumorigenesis in response to CR infection in outbred mice while inflammation and/or colitis in the inbred mice, driven by the expression of distinct cytokines/chemokines, is regulated by activation of the MEK/ERK/NF-κB pathways [39]. It was shown previously that TLR4 signaling contributes to inflammation induced by CR [40]. Based on the recent findings that TLR4 antagonizes β-catenin-induced cell proliferation in the small intestine but not in the colon, we hypothesized that β-catenin and not necessarily NF-κB will dictate the colonic crypt hyperplastic response following CR infection in mice deficient for *Tlr4*. This hypothesis was tested in the current study.

## Results

**Figure 1:**
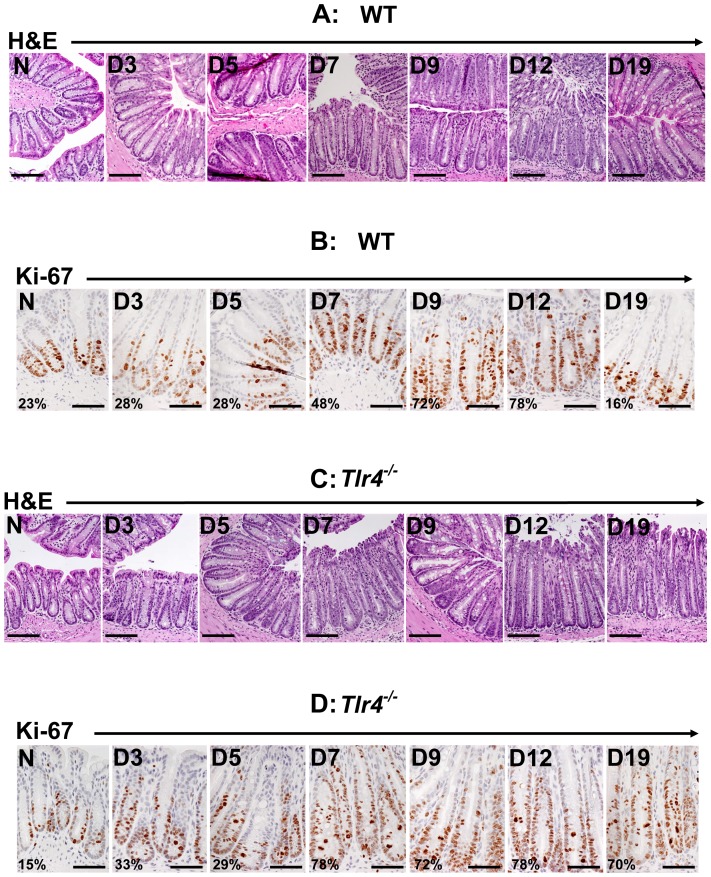
Effect of *Citrobacter rodentium* (CR) infection on gross morphology. H&E staining in the paraffin-embedded sections prepared from the distal colons of uninfected normal (N) and CR infected (days 3–19) C57Bl/6 (A) and *Tlr4^−/−^* (C) mice, respectively. Scale bar, 100 µm. Crypt hyperplasia as measured by Ki-67 staining. Immunohistochemical labeling of Ki-67 as a marker of proliferation in paraffin-embedded sections prepared from the distal colons of uninfected normal (N) and CR-infected (days 3–19) C57Bl/6 (B) and *Tlr4^−/−^* (D) mice, respectively. Percentages represent percent cells positive for Ki-67. Scale bar = 100 µm (n = 3 independent experiments).

Infection of C57Bl/6 wild type mice with *Citrobacter rodentium* (CR) elicited a predictable response in the distal colon: gross thickening accompanied by hyperplasia and significant increase in crypt length between days 7 to 12 post-infection. The crypt length however, plateaued between days 12 to 19 (**Fig. 1A**). To determine if changes in epithelial cell proliferation contributed towards differences in crypt lengths, we next stained colonic sections for Ki-67 as a marker for proliferation. Representative sections from the distal colons of uninfected normal (N) mice and from days 3 to 19 post-infected mice are shown in **Fig. 1B**. In normally proliferating crypts, only cells at the base exhibited nuclear staining (**Fig. 1B**). Between days 3–7 post-infection, a gradual increase in Ki-67 staining was recorded which peaked by day 9 before tapering off between days 12–19 (**Fig. 1B**).

Infection of *Tlr4^−/−^* mice with CR elicited a much more profound response both in terms of gross morphology and crypt epithelial cell proliferation (**Fig. 1C, D**). Interestingly, neither the crypt length nor the cell proliferation in response to CR infection decreased between days 7–19. On the contrary, Ki-67 staining even at day 19 (**Fig. 1D**) was significantly higher than the wild type counterpart (see **Fig. 1B**) suggesting a more aggressive response to infection in the absence of functional TLR4.

**Figure 2:**
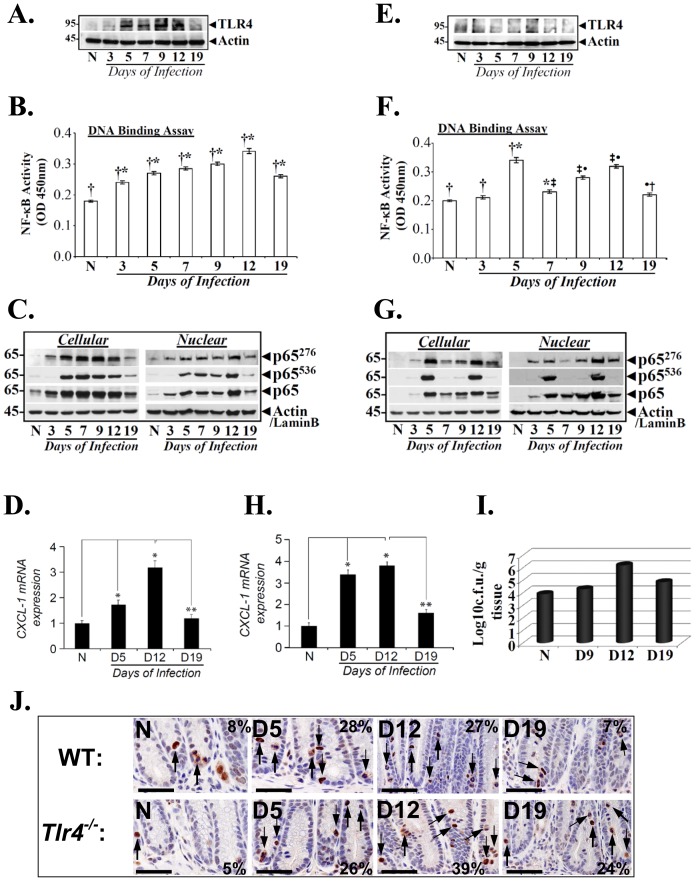
TLR4-dependent and independent mechanism of NF-κB activation during TMCH. Relative levels of TLR4 in the cellular extracts prepared from the isolated crypts of uninfected normal (N) and CR infected (days 3–19) C57Bl/6 (A) and *Tlr4^−/−^* (E) mice, respectively. Actin was used as internal control. Measurement of NF-κB activity in the colonic crypts of C57Bl/6 and TLR4^−/−^ mice. NFκB-p65 activity in the nuclear extracts prepared from the uninfected normal (N) and days 3–19 post infected C57Bl/6 (B) and *Tlr4^−/−^* (F) mice was examined by utilizing TransAM NF-κB p65 Chemi Transcriptional Factor assay kit from Active Motif. ^†^*, p<0.05 vs. N; *^‡^, p<0.05 vs. day 5; ^‡•,^ p<0.05 vs. day 7; ^•†^, p<0.05 vs. day 12 (n = 3 independent experiments). Phosphorylation status of p65 subunit in the colonic crypts of C57Bl/6 and *Tlr4^−/−^* mice. Relative levels of phosphorylated (p65^276^/p65^536^) and total p65 subunit in the cellular and nuclear extracts prepared form the uninfected normal (N) and days 3–19 post infected C57Bl/6 (C) and *Tlr4^−/−^* (G) mice were determined by Western blotting with moiety-specific antibodies. Actin or LaminB were loading controls. CXCL-1/KC expression in the colonic crypts of C57Bl/6 and *Tlr4^−/−^* mice. Expression of CXCL-1/KC mRNA isolated from the crypts of uninfected normal (N) and days 3–19 post infected C57Bl/6 (D) and TLR4^−/−^ (H) mice respectively, was measured as readout for NF-κB activity, via real-time RT-PCR. *, p<0.05 vs. N; **, p<0.05 vs. day 12, (n = 3 independent experiments). I. Bacterial counts correlate with NF-κB activation in *Tlr4^−/−^* mice. Distal colonic homogenates, prepared from the uninfected and CR-infected (days 9–19) *Tlr4^−/−^* mice were plated on McConkey agar and incubated at 37°C. Pink *Citrobacter rodentium* colonies were counted and plotted as log values (n = 3 independent experiments). J. Immunohistochemical staining of p65 subunit phosphorylated at Ser-276 *in vivo*. Paraffin embedded sections prepared from the distal colons of uninfected normal (N) and days 5, 12 and 19 post-infected wild type C57Bl/6 (WT) and *Tlr4^−/−^* mice were stained with antibody specific for NFκB-p65 phosphorylated at Ser-276 and were analyzed with light microscopy. Percentages represent percent cells positive for p65^276^ staining. (Scale bar = 75 µm; n = 3 independent experiments).

We have recently shown that NF-κB activation in response to CR infection involves signaling via TLR4 [41]. To determine if CR infection affected changes in TLR4 levels and to definitively characterize TLR4’s role in NF-κB activation during TMCH, we began by examining sequential changes in TLR4 in the colonic crypts of C57Bl/6J mice. As shown in **Fig. 2A**, TLR4 levels started to increase by day 3 and peaked between days 5–12 compared to uninfected control before declining at day 19. During measurement of NF-κB activity in the crypt nuclear extracts, a sequential increase was recorded between days 3–12 before activity declined at day 19 (**Fig. 2B**). We next investigated the phosphorylation status of p65-NF-κB to determine whether post-translational modification of this subunit contributed towards sustained activation of NF-κB following CR infection. When we utilized antibodies specific for Serines-276 (p65^276^) and −536 (p65^536^) phosphorylation of p65 subunit, relative levels of both crypt cellular and nuclear p65^276^ and p65^536^ along with total p65 increased significantly between days 3–12 compared with uninfected control while relative levels of both moieties declined at day 19 (**Fig. 2C**) and correlated with NF-κB activity and expression of its downstream target CXCL-1/KC at these time points (**Fig. 2D**). The bar graph in **Fig. 2D** represents the ratio of CXCL-1 normalized to actin from three independent experiments. These studies corroborate well with other murine models of IBD [42] suggesting that increases in TLR4 may be critical in regulating NF-κB activity during CR-induced crypt hyperplasia.

When *Tlr4^−/−^* mice were infected with CR for 3–19 days, there was no detectable TLR4 in the crypts of these mice as expected (**Fig. 2E**). During measurement of NF-κB activity in the crypt nuclear extracts, we did not observe any change at day 3 post-infection suggesting that absence of TLR4 apparently affected early activation of NF-κB in response to CR infection. At days 5 and 12 however, a bi-phasic activation of NF-κB was recorded followed by a declining trend at day 19 (**Fig. 2F**). Western blots with antibodies to p65^276^ and p65^536^ at days 5 and 12 dramatically correlated with NF-κB activation kinetics while changes in relative cellular and nuclear levels at other time points were less significant (**Fig. 2G**). Total p65 on the other hand, exhibited a sequential increases between days 5–12 before declining at day 19 (**Fig. 2G**). The changes in NF-κB activity at days 5, 12 and 19 correlated well with CXCL-1/KC kinetics (**Fig. 2H**). The bar graph in **Fig. 2H** represents the ratio of CXCL-1 normalized to actin from three independent experiments. These studies clearly suggest that while early NF-κB activation in response to CR infection of *Tlr4^−/−^* mice requires signaling via TLR4, later time points exhibit a TLR4-independent mechanism of NF-κB activation in response to CR infection. Given that TLR4 plays an important role in protective immunity wherein, it limits bacterial dissemination to mesenteric lymph nodes and peripheral organs such as liver and spleen, we next measured bacterial counts in the distal colons of uninfected and CR-infected *Tlr4^−/−^* mice. As depicted in **Fig. 2I**, we did not measure any appreciable increase in bacterial counts at day 9 post-infection. At day 12, however, significant increase in bacterial counts compared to control, were recorded followed by a decline at day 19 (**Fig. 2I**). Whether the increase in bacterial counts at day 12 also represents bacterial translocation to subepithelial compartments is currently being investigated. Next, we examined the extent of crypt labeling for p65^276^ and tried to correlate with changes in NF-κB activity and crypt hyperplasia utilizing tissue sections prepared from uninfected and days 5, 12 and 19 post-CR infected mouse distal colons from wild type C57Bl/6 and *Tlr4^−/−^* mice. As depicted in **Fig. 2J**, p65^276^ labeling in sections from uninfected mouse distal was restricted to the base of the crypt. At days 5 and 12, p65^276^ labeling in the crypts of C57Bl/6 mice increased significantly (**Fig. 2J**) and correlated with changes in NF-κB activity recorded at these time points (see **Fig. 2B**) while p65^276^ labeling decreased significantly at day 19 (**Fig. 2J**). Interestingly, p65^276^ labeling at day 5 and particularly at days 12 and even 19 in *Tlr4^−/−^* mice was significantly higher than the wild type counterpart and correlated with NF-κB activity at these time points (see **Fig. 2F**) and crypt hyperplasia (**Fig. 1**). **Figure S1** further shows evidence of significant p65^276^ labeling at days 9, 12 and 19 compared to uninfected control which correlates with gross changes in the colonic mucosa. Thus, TLR4 requirement is not absolute for NF-κB activation in response to CR infection.

**Figure 3:**
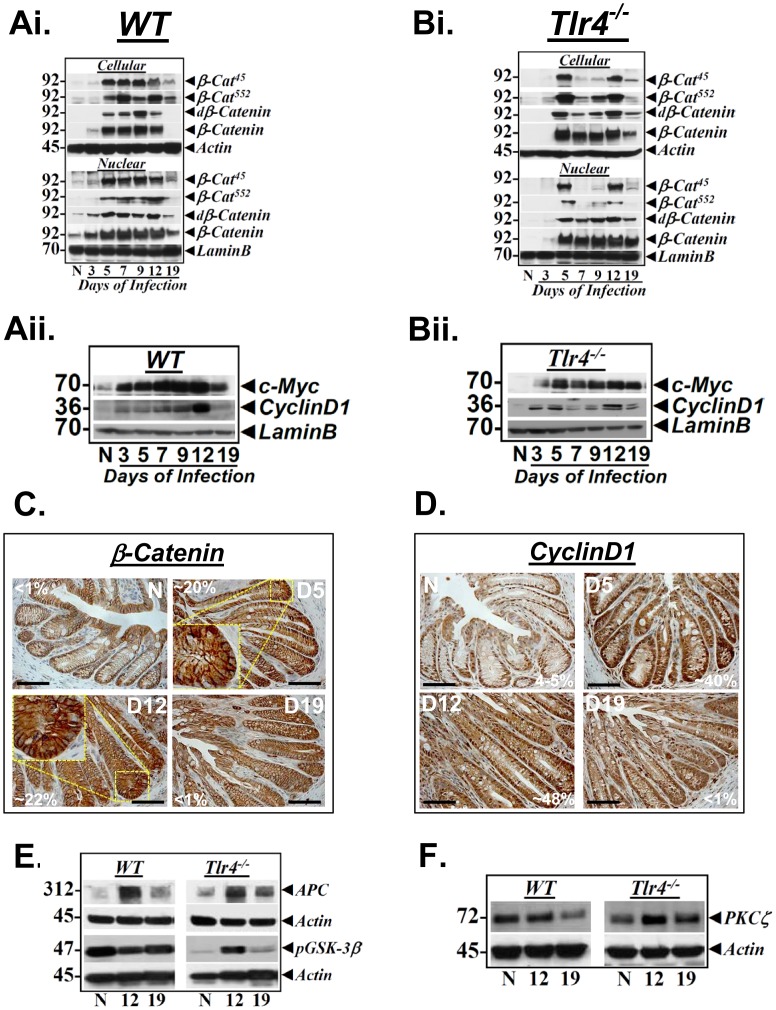
Effect of CR infection on Wnt/β-catenin signaling *in vivo*. Relative levels of phosphorylated (β-Cat^45^/β-Cat^552^), de-phosphorylated (dβ-Catenin) and total β-catenin in the cellular and nuclear extracts prepared form the uninfected normal (N) and days 3–19 post infected wild type (WT) C57Bl/6 (Ai) and *Tlr4^−/−^* (Bi) mice were determined by Western blotting with moiety-specific antibodies. Actin or LaminB were loading controls. Downstream targets of Wnt/β-catenin signaling are upregulated in response to CR infection. Relative levels of cyclinD1 and c-Myc in the nuclear extracts prepared from the uninfected normal (N) and days 3–19 post infected wild type (WT) C57Bl/6 (Aii) and *Tlr4^−/−^* (Bii) mice were determined by Western blotting. LaminB was used as loading control. C and D. Immuno-staining for β-catenin and cyclinD1 in *Tlr4^−/−^* mice. Paraffin embedded sections prepared from the distal colons of uninfected normal (N) or 5, 12 and 19-days post-CR infected *Tlr4^−/−^* mice were stained for β-catenin (C) or cyclinD1 (D) respectively. Inset shows diffused nuclear/peri-nuclear staining. Percentages represent percent nuclear positivity for indicated proteins. Scale bar: 50 µm; n = 2 independent experiments. E and F. Increases in β-catenin are not due to functional inactivation of APC protein. Western blots showing relative levels of APC (E), GSK-3β phosphorylated at Ser-9 (E) and PKCζ (F) in the cellular crypt extracts prepared form the uninfected normal (N) and days 12 and 19 post infected wild type (WT) C57Bl/6 and *Tlr4^−/−^* mice, respectively (n = 3 independent experiments).

Next, we examined the contribution of β-catenin towards crypt hyperplasia in response to CR infection in the wild type and *Tlr4* ^−/−^ mice, respectively. We began by measuring moiety-specific phosphorylation of β-catenin using specific antibodies. In the C57Bl/6 wild type mice, cellular and nuclear levels of Ser^45^-phosphorylated β-catenin (β-Cat^45^) increased between days 5–12 post-infection compared to uninfected control before declining at day 19 (**Fig. 3A** **i**) and correlated with gross changes in hyperplasia. Increases in Ser^45^-phosphorylation when normalized to total β-catenin levels however exhibited only subtle enhancement in phosphorylation intensity suggesting that increases in β-Cat^45^ may be due to a proportional increase in overall β-catenin abundance (**Fig. 3A** **i**). Similarly, relative levels of β-cat^552^ as well as de-phospho-β-catenin (dβ-Cat), both known to represent active β-catenin, increased significantly in both the cellular and nuclear extracts between days 5–12 compared to uninfected control before a rapid decline to basal levels at day 19 (**Fig. 3A** **i**). As before, the increases in these species when compared to changes in total β-catenin were subtle. The alterations in various β-catenin species correlated with relative levels of downstream targets c-myc and cyclinD1 (**Fig. 3A** **ii**). In *Tlr4^−/−^* mice, increases in cellular and nuclear levels of β-Cat^45^ and β-Cat^552^ at days 5 and 12 compared to uninfected control (**Fig. 3B** **i**), were mirror images of changes recorded in phosphorylated p65 at these time points (see **Fig. 2G**) while changes at other time points were less significant. In contrast, while the cellular levels of dβ-Cat followed the β-Cat^45^ and β-Cat^552^ pattern, nuclear levels (**Fig. 3B** **i**) were similar to those recorded in the crypts of wild type C57Bl/6 mice (see **Fig. 3A** **i**) except that the levels even at day 19 were significantly higher than uninfected control. The increases in these species were apparently a reflection of an overall increase in total β-catenin at these time points but unlike wild type crypts wherein, the total β-catenin returned to baseline at day 19, total β-catenin in *Tlr4^−/−^* crypts remained elevated than uninfected control (**Fig. 3B** **i**). When we measured downstream targets, both c-myc and cyclinD1 exhibited kinetics similar to that recorded in wild type crypts except that the levels even at day 19 were still higher than uninfected control (**Fig. 3B** **ii**). These changes correlated well with persistence of crypt hyperplasia at this time point (see **Fig. 1D**). The changes in cellular/nuclear β-catenin and cyclinD1 in *Tlr4^−/−^* mice were further validated via immunohistochemistry. As depicted in **Figure 3C**, a significant increase in diffused cytosolic, nuclear and peri-nuclear staining for β-catenin was recorded at days 5 and 12 compared to uninfected control while nuclear staining declined at day 19. Similar staining pattern for cyclinD1 (**Fig. 3D**) coincided with Western blotting data (see **Fig. 3B** **ii**).

Cytosolic levels of free β-catenin are regulated by APC protein. We have shown previously that increases in cellular/nuclear levels of β-catenin in response to CR infection were neither due to mutational activation of β-catenin or loss of functional APC protein [35]–[37], [43]. In the current study, relative increases in cellular levels of p312 were recorded at day 12 followed by a decline at day 19 in the colonic crypts of both the wild type and *Tlr4^−/−^* mice suggesting that increases in β-catenin at day 12 were not due to loss of wild type APC (**Fig. 3E**). At the same time and as shown by us previously [35], increases in β-catenin were probably due to GSK-3β’s phosphorylation at Ser-9 which results in its inactivation. These changes in phospho-GSK-3β were more pronounced in the colonic crypts of *Tlr4* ^−/−^ mice compared to the wild type counterpart (**Fig. 3E**). Finally, consistent with the earlier finding [44], we observed significant increases in PKCζ, the kinase that phosphorylates Ser-9 of GSK3β [35], at day 12 post-infection compared to uninfected control in the colonic crypts of *Tlr4^−/−^* mice suggesting (but certainly not proving) PKCζ-mediated inactivation of GSK-3β at this time point while changes in wild type mice were less significant (**Fig. 3F**).

### Differential Regulation of β-catenin and NF-κB Pathways *in vitro*

**Figure 4:**
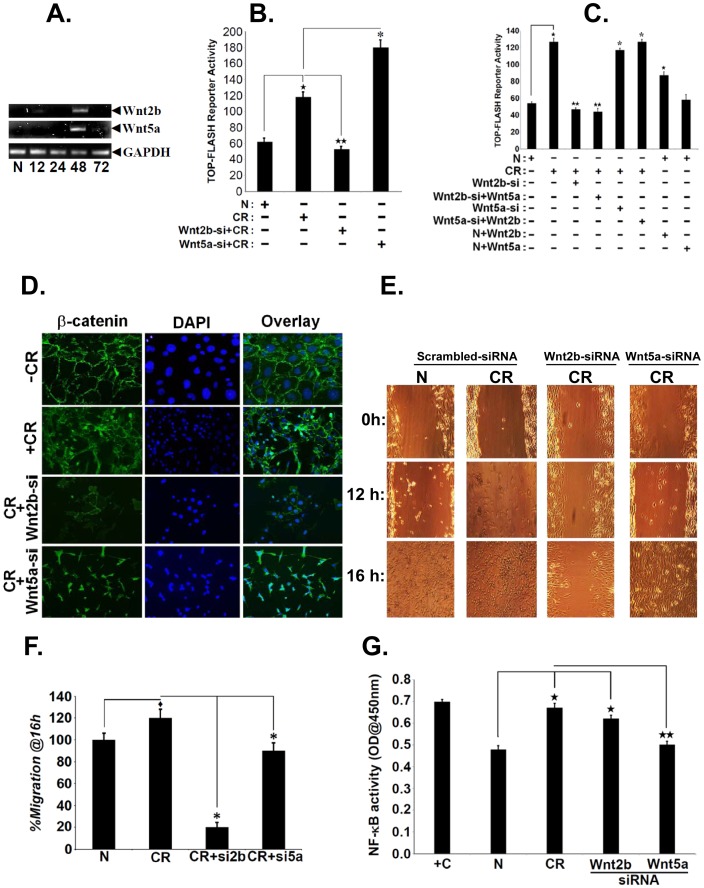
Evidence of β-catenin and NF-κB interplay *in vitro*. A. Measurement of Wnt2b and Wnt5a expression in YAMC (Young Adult Mouse Colon) cells *in vitro*. YAMC cells (5×10^5^) were either uninfected (N) or infected with CR at 90∶1 MOI for 3 hr. Cells were washed thoroughly to remove bacteria and incubated in fresh medium containing antibiotics for indicated period of time. Total RNA was examined for the expression of Wnt2b and Wnt5a via RT-PCR. GAPDH was used as loading control. B. Effect of Wnt2b knockdown on reporter activity. YAMC cells were transiently transfected with TOPflash plasmid and with siRNA specific to Wnt2b and Wnt5a, respectively. After 24 h, cells were infected with CR at 90∶1 MOI for 3 h, washed to remove bacteria followed by measurement of reporter activity at 48 h using Renilla luciferase as internal control (^★^, p<0.05 vs. N; ^★★^, p<0.05 vs. CR; *, p<0.05 vs. CR; n = 3 independent experiments). C. Effect of Wnt2b and Wnt5a addition on reporter activity. YAMC cells, following transient transfection for 24 h with TOPflash plasmid and with siRNA specific to Wnt2b and Wnt5a, respectively. After 24 hr, cells were incubated with purified Wnt2b or Wnt5a, infected with CR at 90∶1 MOI for 3 h, washed to remove bacteria followed by measurement of reporter activity at 48 h using Renilla luciferase as internal control (^★^, p<0.05 vs. N; ^★★^, p<0.05 vs. CR; *, p<0.05 vs. CR; n = 3 independent experiments). D. Effect of Wnt2b and Wnt5a knockdown on β-catenin nuclear localization. YAMC cells were transfected with siRNA specific to Wnt2b and Wnt5a, respectively for 24 h. Cells were subsequently treated with control media or with CR (<@email>@90<@/email>∶1 MOI) for 3 hr followed by washing to remove bacteria. At 24 hr post-infection, cells were stained with antibody for β-catenin while nuclei were stained with DAPI. E. Effect of Wnt2b and Wnt5a knockdown on wound healing. YAMC cells were transfected with siRNA specific to Wnt2b and Wnt5a, respectively for 24 h. Cells were subsequently treated with control media or with CR (@90∶1 MOI) for 3 hr followed by washing to remove bacteria. At 48 h, cells were wounded with a plastic pipette tip. After removing debris and adding fresh media, cell migration was followed for 16 h. Figure 4F is a representative bar graph showing percent migration at 16 h (♦, p<0.05 vs. N; *, p<0.05 vs. CR; n = 3 independent experiments). G. Effect of Wnt2b and Wnt5a knockdown on NF-κB activity. YAMC cells were transfected with siRNA specific to Wnt2b and Wnt5a, respectively for 24 h. Cells were subsequently treated with control media or with CR (@90∶1 MOI) for 3 hr followed by washing to remove bacteria. At 48 h, cells were lysed and NF-κB activity in the nuclear extracts was examined by utilizing TransAM NF-κB p65 Chemi Transcriptional Factor assay kit from Active Motif (^★^, p<0.05 vs. N; ^★★^, p<0.05 vs. CR; n = 3 independent experiments).

So far, we have seen that both increases in NF-κB and β-catenin signaling following CR infection coincide with colonic crypt hyperplasia in wild type as well as *Tlr4^−/−^* mice, respectively. To investigate if an interplay between the two pathways exists, we opted for *in vitro* studies to focus on two proteins, Wnt2b and Wnt5a that activate Wnt/β-catenin and NF-κB pathways, respectively [45], [27]. Semi-quantitative PCR revealed significant increases in both Wnt2b and Wnt5a expression in YAMC cells at 48 h post-CR infection compared to uninfected control (**Fig. 4A**). During measurement of β-catenin/Tcf4-dependent TOP-flash reporter activity, CR infection induced a 2-fold increase in reporter activity at 48 h post-infection (**Fig. 4B**). In response to siRNA-mediated Wnt2b knockdown, reporter activity decreased significantly and was not rescued with CR infection (**Fig. 4B**). Wnt5a knockdown on the other hand, resulted in an increase in reporter activity which was further enhanced following CR infection (**Fig. 4B**). Next we examined the effect of adding purified Wnt2b and Wnt5a proteins on reporter activities. CR infection compared to uninfected control increased the reporter activity by ∼2-fold as described before (**Fig. 4C**). Si-RNA to Wnt2b almost completely attenuated the reporter activity while addition of purified Wnt5a did not rescue the inhibitory effects (**Fig. 4C**). Si-RNA to Wnt5a had no effect on the CR-induced reporter activity which remained elevated while addition of Wnt2b further enhanced the reporter activity (**Fig. 4C**). To confirm if these changes were mediated by increases in β-catenin, we stained YAMC cells for β-catenin. Immunofluorescent studies revealed predominantly membranous staining for β-catenin in uninfected cells (**Fig. 4D**). In contrast, significant nuclear labeling of β-catenin was observed in response to CR infection (**Fig. 4D**). Interestingly, siRNA to Wnt2b almost completely blocked β-catenin nuclear translocation while siRNA to Wnt5a had no effect (**Fig. 4D**). Similarly, during scratch-induced wound assay in the YAMC cells, knockdown of Wnt2b but not Wnt5a significantly blocked β-catenin-dependent wound healing (**Fig. 4E**). **Figure 4F** is a representative bar graph showing percent migration at 16 h. Thus, Wnt2b and not Wnt5a promotes Wnt signaling in response to CR infection. We next examined the effect of siRNA-mediated knockdown of Wnt2b and Wnt5a on NF-κB activity *in vitro*. During the DNA binding assay, NF-κB activity increased significantly in response to CR infection compared to uninfected control while siRNA to Wnt2b had no effect (**Fig. 4G**). On the other hand, siRNA to Wnt5a almost completely attenuated CR-induced NF-κB activity (**Fig. 4G**). Thus, Wnt5a and not Wnt2b regulates NF-κB activity in response to CR infection.

**Figure 5:**
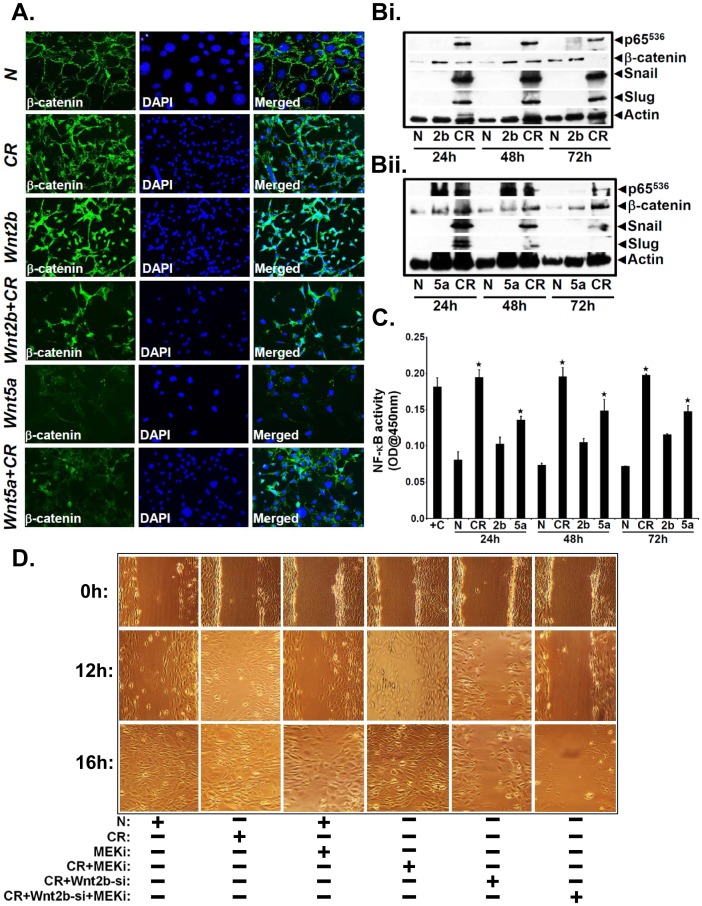
Evidence of differential β-catenin and NF-κB signaling by Wnt ligands *in vitro*. A. YAMC cells were either treated with control media or infected with CR (@90∶1 MOI) for 3 h followed by washing to remove bacteria. Uninfected or CR infected cells were incubated with purified Wnt2b or Wnt5a for 24 hr followed by immuno-staining for β-catenin. Nuclei were stained with DAPI (n = 3 independent experiments). B. Effect of Wnt2b and Wnt5a addition on cellular levels of β-catenin and NF-κB. YAMC cells described in A were incubated with purified Wnt2b or Wnt5a for 24, 48 and 72 hr followed by Western blotting with antibodies for: p65 subunit phosphorylated at Ser-536 (p65^536^), β-catenin, Slug and Snail, respectively. Actin was used as loading control. C. Effect of Wnt2b and Wnt5a addition on NF-κB activity. YAMC cells described in B were subjected to nuclear extraction followed by measurement of NF-κB activity utilizing TransAM NF-κB p65 Chemi Transcriptional Factor assay kit from Active Motif (^★^, p<0.05 vs. N; n = 3 independent experiments). D. Effect of MEK inhibitor (MEKi) on wound healing. YAMC cells were transfected with control or Wnt2b-specific siRNA for 24 h followed by incubation with MEKi. At 48 h, cells were wounded with a plastic pipette tip and cell migration was followed for 12–16 h (n = 3 independent experiments).

As proof-of-principle, we further characterized the role(s) of purified Wnt2b and Wnt5a proteins in modulating the Wnt and NF-κB pathways *in vitro*. During immunostaining, β-catenin remained membrane bound in uninfected cells while a predominant nuclear staining was recorded in CR infected cells (**Fig. 5A**), as described before (see **Fig. 4D**). Interestingly, purified Wnt2b was more potent than even CR infection in facilitating β-catenin’s nuclear import while a combination of Wnt2b+CR did not necessarily yield an additive response (**Fig. 5A**). Addition of purified Wnt5a on the other hand, did not affect either β-catenin levels or nuclear translocation while a combination of Wnt5a+CR restored β-catenin’s nuclear localization (**Fig. 5A**). During a time course with purified proteins, addition of Wnt2b led to significant increases in relative levels of nuclear β-catenin at 24 hr and the levels persisted until 72 hr post-addition (**Fig. 5B** **i**). These increases were either at par or even higher than those recorded with CR infection (**Fig. 5B** **i**). Interestingly, we did not observe any detectable levels of either p65 phosphorylated at Ser-536 (p65^536^) or β-catenin’s downstream targets Slug and Snail following addition of purified Wnt2b while CR infection alone promoted increases in these proteins at indicated times (**Fig. 5B** **i**). Wnt5a addition on the other hand, led to significant increases in relative levels of nuclear p65^536^ at 24 hr which persisted until 48 hr before declining at 72 hr similar to those recorded with CR infection alone while Wnt5a only had nominal effect on β-catenin levels at these time points (**Fig. 5B** **ii**). Similar to Wnt2b, Wnt5a did not affect Slug and Snail expression at either time point (**Fig. 5B** **ii**). Next, during measurement of NF-κB activity, CR infection compared to uninfected control, caused a reproducible increase in NF-κB activity at 24 hr which persisted until 72 hr (**Fig. 5C**). Following addition of Wnt5a, a significant increase in NF-κB activity was recorded at 24 hr which persisted until 72 hr while addition of Wnt2b had subtle effect on NF-κB activity (**Fig. 5C**). Finally, based on our previous report regarding activation of MEK/ERK pathway following CR infection in the colonic crypts [41], we performed scratch-induced wound assay in YAMC cells in the presence or absence of MEK inhibitor PD98059 (MEKi) to understand if MAPK signaling may be contributing towards CR+Wnt2b-induced wound healing. As depicted in **Figure 5D**, CR infection compared to uninfected cells, caused almost complete wound closure between 12–16 h (**Fig. 5D**). Treatment of uninfected cells with MEKi only caused a subtle failure in cell migration while CR infection of MEKi-treated cells neutralized the inhibitory effect (**Fig. 5D**). Si-RNA to Wnt2b significantly blocked CR-induced cell migration (**Fig. 5D**) as described earlier (see **Fig. 4E**). Interestingly, a combination of siRNA-Wnt2b+MEKi almost completely abrogated CR-induced cell migration (**Fig. 5D**) suggesting that Wnt2b/MEK cross-talk may be required for CR-induced cell migration. **Figure S2A** is a representative bar graph showing percent migration at 16 h. Taken together, these studies clearly implicate both canonical and non-canonical Wnt pathways in the differential regulation of β-catenin and NF-κB in response to CR infection.

### Blocking β-catenin *in vivo* Abrogates Crypt Hyperplasia and Reduces Tumorigenesis

**Figure 6:**
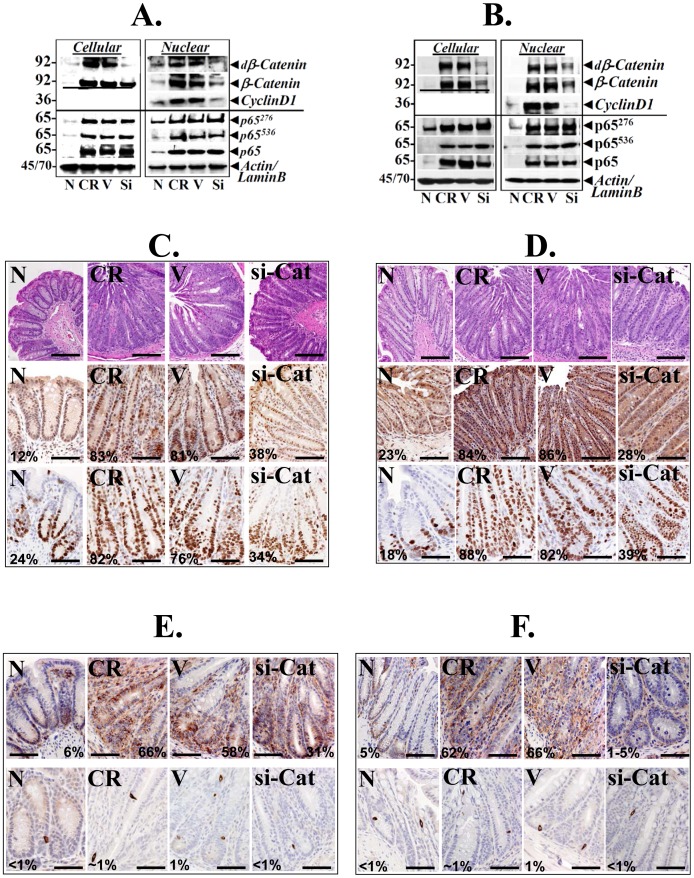
Signaling via β-catenin is integral to crypt hyperplasia. CR-infected wild type C57Bl/6 (A) and *Tlr4^−/−^* (B) mice either received vehicle (V) or ip injections of nanoparticle-encapsulated β-catenin siRNA (Si) every alternate day for 10 days. Colonic crypts were isolated and subjected to cellular or nuclear extract fractionation followed by Western blotting for indicated proteins. Actin and LaminB were used as loading controls. C–F. Paraffin embedded sections prepared from the distal colons of uninfected normal (N), CR infected (CR), CR infected+vehicle treated (V) or CR infected+β-catenin siRNA treated (si-Cat) mice were stained with: C and D. H&E for gross morphology (upper panel), β-catenin phosphorylated at Ser-552 to detect active β-catenin (middle panel) and Ki-67 for proliferation (lower panel); E and F. CD44 (upper panel) and Dclk1 (lower panel) were also stained to determine the effect of β-catenin knockdown on stem cells. Percentages represent percent cells positive for indicated markers. Scale bar: 125 µm; n = 2 independent experiments.

**Figure 7:**
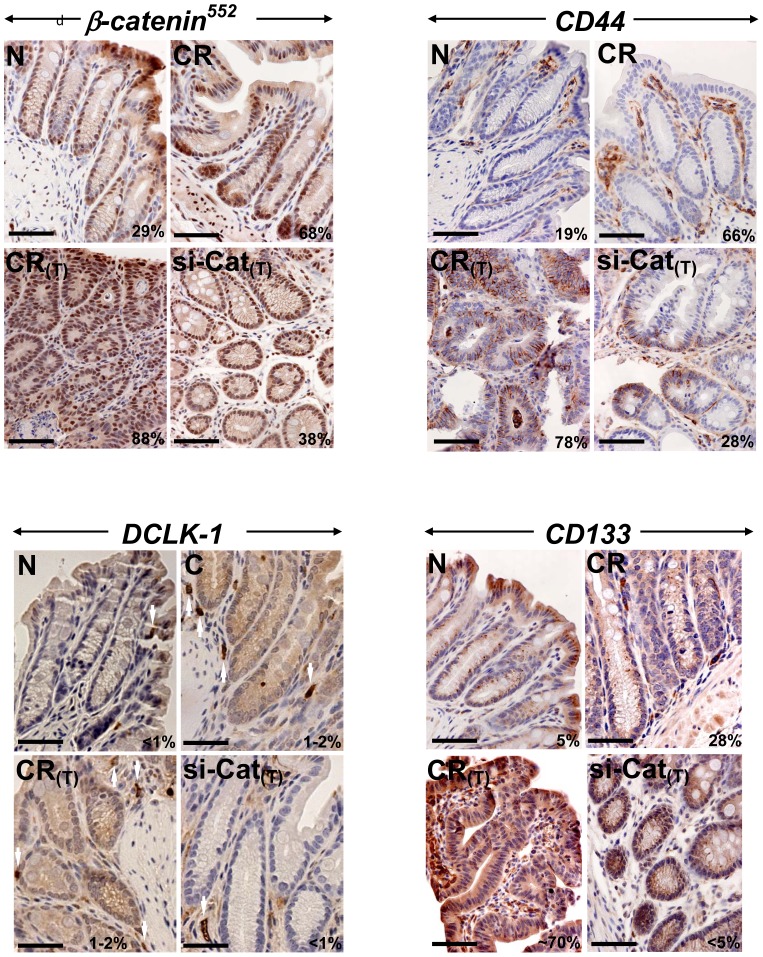
Effect of β-catenin knockdown on tumorigenesis in *Apc* ^Min/+^ mice. Representative photomicrographs of paraffin embedded sections prepared from the distal colons of uninfected normal (N), CR-infected at day 12, CR-infected mice at 3 months of age with tumor (CR_T_) and CR_T_ mice treated with nanoparticle-encapsulated β-catenin siRNA (si-Cat_(T)_). Sections were stained with antibodies for: β-catenin phosphorylated at Ser-552 (β-catenin^552^) and cancer stem cell markers CD44, Dclk1 and CD133, respectively. Percentages represent percent cells positive for indicated markers. Scale bar: 50 µm; n = 2 independent experiments.

Our next aim was to understand the interplay between the β-catenin and NF-κB pathways *in vivo* in response to CR infection in the wild type (WT) mice and mice deficient for TLR4. In response to either CR infection or CR infection+vehicle treatment of either strain, significant increases in both active and total β-catenin was observed in the crypt cellular and nuclear extracts along with increases in its downstream target cyclinD1, compared to uninfected control (**Fig. 6A, B**). When WT or *Tlr4* ^−/−^ mice were treated with nanoparticle-encapsulated siRNA to β-catenin (si-β-Cat), we observed almost complete loss of nuclear β-catenin with concomitant decreases in cyclinD1 (**Fig. 6A, B**). As expected, si-β-Cat treatment had no effect on active (p65^276^, p65^536^) or total NF-κB-p65 which remained elevated in CR infected crypt cellular and nuclear extracts (**Fig. 6A, B**). Interestingly, si-β-Cat treatment significantly blocked β-Cat^552^ nuclear staining concomitant with decreases in cell proliferation as was determined by reduced Ki-67 staining in treated samples without grossly affecting the mucosa (**Fig. 6C, D**). To see if downstream targets of Wnt/β-catenin signaling and putative markers of stem cells were affected, we next examined the effect of si-β-Cat treatment on CD44 and Dclk1 staining in the two sets of mice. In response to either CR infection or CR infection+vehicle treatment, staining for both CD44 and Dclk1 increased in the colonic crypts (**Fig. 6E, F**). Following si-β-Cat treatment, staining for both CD44 and Dclk1 were reduced to baseline (**Fig. 6E, F**). Interestingly, the effect of si-β-Cat treatment on CD44 was much more pronounced in sections prepared from *Tlr4^−/−^* mice compared to wild type counterpart (**Fig. 6F**). We have shown recently that functional cross-talk between Notch and NF-κB pathways regulate crypt hyperplasia and/or tumorigenesis in response to CR infection [38]. We therefore examined the effect of si-β-Cat treatment on cancer stem cell markers in *Apc* ^Min/+^ mice to see if tumorigenesis is affected. si-β-Cat treatment to *Apc* ^Min/+^ mice significantly attenuated increases in β-Cat^552^, CD44, Dclk1 and CD133 that halted the growth of mutated crypts (**Fig. 7**) without affecting NF-κB-p65^276^ signaling (data not shown). Thus, β-catenin and not necessarily NF-κB regulates crypt hyperplasia and tumorigenesis in response to CR infection.

### Evidence of β-catenin and not NF-κB-induced Crypt Hyperplasia in CAST.11M Mice

**Figure 8:**
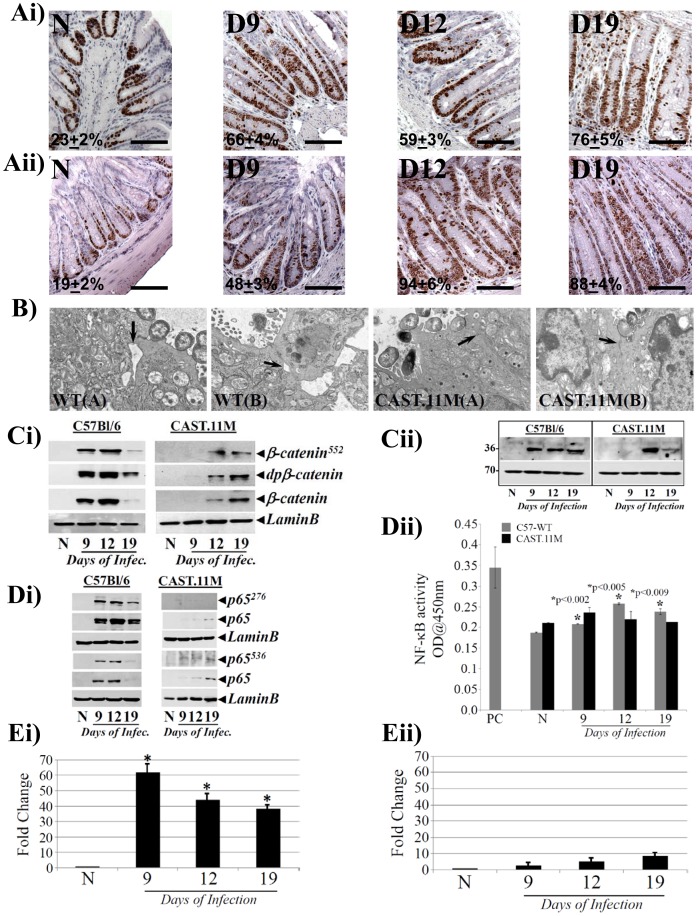
B6.CAST.11M mice exhibit a hyperplastic but a muted inflammatory response to CR infection. A. Representative photomicrographs of paraffin embedded sections prepared from the distal colons of uninfected normal (N) and days 9–19 post-CR infected C57Bl/6 (upper panel) or CAST.11M (lower panel) mice and stained for Ki-67 (percentages represent percent cells positive for Ki-67). Scale bar: 50 µm; n = 3 independent experiments. B. Electron Microscopy. Distal colonic fragments from CR infected wild type C57Bl/6 (WT) or CAST.11M mice were subjected to transmission electron microscopy to detect disruption in tight junctions (arrows). Two representative images are shown for each group of mice. C. Relative levels of phosphorylated (β-Cat^552^), de-phosphorylated (dpβ-Catenin) and total β-catenin (Ci) and cyclinD1 (Cii) in the nuclear extracts prepared form the uninfected normal (N) and days 9, 12 and 19 post infected wild type C57Bl/6 and B6.CAST.11M mice, were determined by Western blotting with moiety-specific antibodies. LaminB was used as loading control. Di. Nuclear extracts prepared from the distal colonic crypts of groups of mice described in C were probed with antibodies for: p65^276^, p65^536^ and total p65, respectively. LaminB was used as loading control. Dii. Measurement of NF-κB activity. NF-κB-p65 activity in the nuclear extracts prepared from the groups of mice described in C was measured by utilizing TransAM NF-κB p65 Chemi Transcriptional Factor assay kit from Active Motif. E. CXCL-1/KC expression in the colonic crypts. Expression of CXCL-1/KC mRNA isolated from the distal colonic crypts of C57Bl/6 (Ei) or CAST.11M (Eii) mice was measured as readout for NF-κB activity, via real-time RT-PCR. *, p<0.05 vs. N; n = 3 independent experiments.

Castaneus mice contain a CAST/Ei region on chromosome 11 (CAST.11M) on an otherwise C57BL/6 genetic background. Genetic variation within an 8.3 Mb region on mouse chromosome 11 controls host response to anthrax lethal toxin and resistance to infection by the Sterne strain of *Bacillus anthracis* [46], [47]. We compared these mice with wild type C57Bl/6 to determine if differences exist in the extent of β-catenin and NF-κB activation and associated crypt hyperplasia in these mice in response to CR infection. Following CR infection, C57Bl/6 mice exhibited significant crypt hyperplasia as revealed by Ki-67 staining at day 9 which peaked by day 12 and plateaued by day 19 (**Fig. 8A** **i**). In CAST.11M mice however, Ki-67 staining and crypt lengths at day 12 and particularly at day 19 compared to uninfected controls were 1.5–2 fold higher than those recorded in C57Bl/6 mice at the same time points (**Fig. 8A** **ii**). Transmission electron microscopy revealed no difference in bacterial binding at the luminal surface in either strain (**Fig. 8B**); yet, C57Bl/6 mice had significant disruption of the tight junctions (**Fig. 8B**), an elevated serum FITC-Dextran levels (**Fig. S2B**) and recruitment of CD3+ T-cells and F4/80+ macrophages to the sub-epithelial regions compared to CAST.11M mice (**Fig. S3**). Mechanistically, in C57Bl/6 mice, Ki-67 kinetics coincided with increases in nuclear accumulation of β-catenin and its downstream target cyclinD1 at days 9 and 12 compared to uninfected control while the levels declined at day 19 (**Fig. 8C** **i, Cii**). In addition, p65^276^/p65^536^ subunit expression (**Fig. 8D** **i**) and NF-κB activity (**Fig. 8D** **ii**) increased significantly at days 9 and 12 with declining trend at day 19 that correlated with downstream target CXCL-1 expression (**Fig. 8E** **i**). In CAST.11M mice on the other hand, we observed a delayed response wherein, nuclear β-catenin levels did not go up until day 12 with sustained expression at day 19 (**Fig. 8C** **i**) which coincided with cyclinD1 expression (**Fig. 8C** **ii**) and Ki-67 kinetics at these time points. Interestingly, CAST.11M mice did not exhibit any significant increase in phosphorylated/total p65 subunit expression (**Fig. 8D** **i**), NF-κB activity (**Fig. 8D** **ii**), CXCL-1 expression (**Fig. 8E** **ii**) or serum FITC-Dextran levels (**Suppl.** **Fig. 2**) at these time points despite adequate bacterial binding to the colonic mucosa (**Fig. 8B**). Thus, β-catenin can regulate crypt hyperplasia in response to CR infection in the absence of NF-κB signaling.

## Discussion

Wnt/β-catenin and NF-κB are independent pathways involved in the regulation of physiological and pathological effects related to the development, immune function, inflammation, tumorigenesis and frank malignancy. The recent discovery of functional cross-regulation between the two pathways has established complex roles for Wnt/β-catenin and NF-κB signaling in the pathogenesis of various diseases including cancer. Indeed, the mechanisms and biological effects of the cross-regulation of the two pathways remains an area of intense investigation. It is now evident that strategies targeting the cross-regulation between these pathways may be a promising direction for future cancer therapeutics.

Recognition of Gram negative bacterial products such as lipopolysaccharide (LPS) by toll like receptor-4 (TLR4) and subsequent activation of NF-κB not only activates host defense against pathogenic bacteria, but can also provide mucosal protection. In pathological situations however, TLR4 is upregulated in both Crohn’s disease and ulcerative colitis, two components of human IBD, leading to pro-inflammatory signaling via NF-κB [42], [48]. We recently showed that NF-κB activation in response to CR infection was dependent upon signaling via TLR4 [41]. In this study, we observed significant increases in cellular levels of TLR4 in the colonic crypts of CR infected mice suggesting critical role(s) for TLR4 in transducing signals in response to CR infection. To definitively implicate TLR4 in CR-induced NF-κB activation, we utilized *Tlr4* ^−/−^ mice in the B6 background wherein, initial kinetics of NF-κB activation at day 3 highlighted the need for the presence of TLR4; NF-κB activation at day 5 however, may represent a homeostatic response to counter bacterial infection while NF-κB activation at day 12 which correlated with peak hyperplasia (see **Fig. 1**) may represent an intrinsic pathway activation independent of TLR4. This is in contrast to decreased epithelial proliferation and significant epithelial injury recorded during DSS-induced colitis in *Tlr4^−/−^* mice [42]. These differences may be attributable to the nature of the colitis-inducing agent: CR infection may represent more physiological approach while DSS-induced damage of the epithelial cells may lead to more severe outcome. Whatever the case may be, the issue remains regarding NF-κB activation in the absence of signaling via TLR4. Since cell types such as macrophages and lymphocytes in addition to epithelial cells, express TLR4, it is probable that NF-κB activation at day 12 in these mice may be a result of stromal-epithelial cross-talk wherein, bacterial translocation to the cells of the stroma (e.g., MΦs, lymphocytes etc.) may eventually facilitate NF-κB activation in the crypts via a paracrine pathway. Indeed, we observed a 1.5-fold increase in bacterial counts at day 12 compared to day 9 (see **Fig. 2I**) which may correlate with NF-κB activation at this time point. Whether these increases in bacterial counts also reflect bacterial translocation is currently being investigated. Nevertheless, this is consistent with TLR4’s role in limiting bacterial translocation in a murine model of colitis [42]. Taken together, a combination of TLR4-dependent and independent mechanism may operate in tandem to regulate NF-κB activity in the colonic crypts of *Tlr4^−/−^* mice following CR infection.

In addition to TLR4’s contribution towards NF-κB activation in response to Gram negative bacteria or LPS, TLR4 has also been involved in modulating the Wnt/β-catenin pathway in the intestine, and that over-expression of TLR4 has been found sufficient for priming the intestinal mucosa towards neoplasia. Indeed, Santaolalla R. et al. [49] have recently shown that TLR4 over-expression induces β-catenin phosphorylation in the colon and in cell lines *in vitro*. The same group previously showed that TLR4 signaling in the colon induces epithelial proliferation and protection against apoptosis [50], [51]. Consistent with these findings, we also find significant activation of the Wnt/β-catenin pathway wherein, changes in various species of β-catenin in response to CR infection in C57Bl/6 mice directly correlated with increases in TLR4 levels, downstream targets such as cyclinD1, a number of stem cell-specific marker proteins such as CD44 and Dclk1 and crypt hyperplasia. However, contrary to the above findings, we found even more dramatic changes in β-catenin cellular abundance and downstream target expression in *Tlr4* ^−/−^ mice and these changes directly correlated with increases in crypt hyperplasia in infected mice. A definitive role for β-catenin in CR-induced crypt hyperplasia in *Tlr4* ^−/−^ mice was established by blocking increases in β-catenin *in vivo* which resulted in attenuation of the hyperplastic response. These results clearly contradict the notion that TLR4 is somehow required for activation of the Wnt/β-catenin pathway. Infact, a recent study by Sodhi CP et al. [28] suggested that TLR4 actually inhibits β-catenin and impairs enterocyte proliferation in the small intestine of neonatal mice with experimental necrotizing enterocolitis (NEC). This effect however, was not seen in the colon of the same neonatal mice nor was it found anywhere in the intestine of adult mice. Thus, even though our findings particularly in *Tlr4*-deficient mice corroborate with those described during NEC, the regulation of Wnt/β-catenin signaling via TLR4 seems more complex than what was initially envisioned.

Continuing with our theme of interplay between the Wnt/β-catenin and the NF-κB pathways, an elegant study recently reported that constitutive IKKβ/NF-κB activation strongly synergized with Wnt signaling to promote intestinal tumorigenesis [52]. We showed previously that functional cross-talk between β-catenin and NF-κB was integral to the hyperproliferative effects of progastrin on proximal colonic crypts [53]. In the current study however, we have discovered that despite significant activation of the NF-κB pathway in the distal colons of wild type or *Tlr4^−/−^* mice or in *Apc* ^Min/+^ mice, knockdown of β-catenin significantly impacted both hyperplasia and tumorigenesis in response to CR infection. Since a combination of canonical and non-canonical Wnt signaling regulates the interplay between the Wnt/β-catenin and NF-κB pathways [54], we have further elucidated the roles of two Wnt ligands, Wnt2b and Wnt5a in the differential regulation of these pathways *in vitro*. Based on functional assays with purified proteins, we conclude that Wnt2b but not Wnt5a regulates the Wnt/β-catenin pathway while Wnt5a and not Wnt2b is involved in the activation of the NF-κB pathway, respectively. In previous studies, Wnt2b is shown to be transiently expressed in the primitive streak during gastrulation and has been suggested to primarily play a supportive role in gastrulation and organogenesis [55]. It is upregulated in liver and biliary epithelial cells of patients with primary biliary cirrhosis [56], and in colonic mucosa of patients with inflammatory bowel disease [57]. Wnt2b is also shown to be expressed in several types of human cancer, such as basal cell carcinoma, gastric cancer, breast cancer, head/neck squamous cell carcinoma, cervical cancer and leukemia. More recently, canonical Wnt2/2b signaling is shown to be required for specification of the lung endoderm progenitors in the developing foregut [58]. Similarly, Wnt5a activates both canonical and non-canonical Wnt signaling pathways and is implicated in a variety of cellular processes during development and carcinogenesis. Importantly, the expression of Wnt5a protein is controlled by the NF-κB signaling pathway, which may be implicated as an essential mediator not only for infection, but also for cancer development [54]. While we present evidence of differential β-catenin and NF-κB regulation by the two Wnt ligands in primary colonocytes *in vitro*, studies in new mouse models allowing specific deletion of genes encoding these proteins in the epithelial cells of the colon will be required to address the potential role(s) of these ligands in either crypt hyperplasia or tumorigenesis in response to CR infection.

The dominance of β-catenin-induced crypt proliferation in response to CR infection was further validated in a Castaneus strain (CAST.11M) that exhibits high susceptibility to Anthrax’s lethal toxin (LT) [47] due to a region at chromosome 11 that encodes the LT-responsive CAST/Ei allele of Nlrp1b inflammasome [47]. Interestingly, LT-mediated lethality in mice was found to be independent of TLR4 function [46]. Thus, while it is intriguing to observe a muted NF-κB response in the distal colons of these mice, it is not known whether modifier genes at chromosome 11 are necessarily interfering with NF-κB activation in response to CR infection. Whatever the case may be, the fact that we still observed significant increases in β-catenin in these mice that correlated with crypt hyperplasia further proves our hypothesis that β-catenin and not necessarily NF-κB regulates crypt hyperplasia in response to CR infection. Given that deregulation of the components of the Wnt/β-catenin signaling has been implicated in a wide spectrum of diseases including cancer, our studies further provide a rationale for targeting the Wnt/β-catenin pathway to treat diseases with infectious etiology.

## Materials and Methods

### Mice, CR Infection and Induction of Hyperplasia

This study was carried out in strict accordance with the recommendations in the Guide for the Care and Use of Laboratory Animals of the National Institutes of Health. All protocols were approved by University of Kansas Medical Center Animal Care and Use Committee. All efforts were made to minimize suffering. Male *Helicobacter pylori*-free *Apc* ^Min/+^ and *Tlr4^−/−^* mice were procured from Jackson Laboratory, Bar Harbor, Maine, USA. B6.CAST.11M Castaneus mice were kindly provided by Drs. Aldons J. Lusis and Richard C. Davis (University of California at Los Angeles) and the generation of these mice has been described previously [59]. B6.CAST.11M mice contain a CAST/Ei region in chromosome 11 (∼31.5 Mb to the terminus) on an otherwise C57BL/6J genetic background [59], [60]. All the mice were maintained in a specific pathogen-free (including helicobacter and parvovirus) environment, and generally used between 5 and 6 weeks of age. As control groups, either littermates or WT C57Bl/6J mice of identical background were used. Transmissible murine colonic hyperplasia was induced in *Apc* ^Min/+^, *Tlr4^−/−^* and wild type C57Bl/6J mice by oral inoculation with a 16-h culture of CR (biotype 4280, ATCC, 10^8^CFUs) identified as pink colonies on MacConkey agar, as previously described [34]–[39], [41], [43], [44], [61]–[67]. Age- and sex-matched control mice received sterile culture medium only.

### RNA Extraction, Semi-quantitative and Real-time PCR

Total RNA was extracted from isolated colonic crypts by using TRI Reagent. Following cDNA synthesis using Superscript II and random primers, gene products specific to Wnt-2b (F: 5′-CACCCGGACTGATCTTGTCT-3′; R: 5′-TGTTTCTGCACTCCTTGCAC-3′) and Wnt5a (5′-CTGGCAGGACTTTCTCAAGG-3′; 5′-CTCTAGCGTCCACGAACTCC-3′) were identified by performing semi-quantitative PCR using 1/20 dilution of the cDNA. For each gene product, the amplification cycle number was chosen empirically within the linear range. The PCR products were separated by polyacrylamide gel electrophoresis and visualized by ethidium bromide staining of the gels under UV light. Gel data were recorded with the Bio-Rad FluorS Imaging System, and relative densities of the bands were determined with Quantity One software (Bio-Rad, Hercules, CA). Gene expression was normalized with β-actin expression. Total RNA samples were also subjected to real-time polymerase chain reaction (PCR) by SYBR chemistry (SYBR Green I; Molecular Probes, Eugene, OR) using primers specific for CXCL-1/KC (F: 5′-GCCAATGAGCTGCGCTGTCAATGC-3′; R: 5′-CTTGGGGACACCTTTTAGCATCTT-3′) and GAPDH (F: 5′-AACTTTGGCATTGTGGAAGG-3′; R: 5′-ACACATTGGGGGTAGGAACA -3′). The changes in mRNA were expressed as fold change relative to control with ± SEM value.

### DNA Binding Assay

For DNA binding assay, nuclear extracts were prepared from the distal colonic crypts of uninfected, or days-3, 5, 7, 9, 12 and 19 infected mice, respectively. The young adult mouse colon (YAMC) cells were maintained in RPMI 1640 medium supplemented with 10% fetal bovine serum, 2 mM glutamine, 50 µg/ml gentamicin, 100 units/ml penicillin, 100 µg/ml streptomycin, and 5 units/ml IFNγ in a humidified incubator with 5% CO_2_ at 33°C. YAMC cells (5×10^5^) were transfected with either 100 nmol/L of scrambled siRNA or siRNAs specific for Wnt2b (sc-155356) and Wnt5a (sc-41113) using 2–8 µl of transfection reagent (sc-29528; Santa Cruz Biotechnology Inc., Santa Cruz, CA) for 24 h. CR strain DBS 100 (ATCC cat.# 51459™) was grown under aerophilic conditions on Luria-Bertani (LB) agar plates for 24 h at 37°C and cultured in LB broth O/N at 37°C. RPMI, containing 0.45% glucose was inoculated with a 1∶20 dilution of a standard LB overnight culture and incubated for 2 h at 37°C. Monolayers of 5 10^5^ YAMC cells at ∼50% confluence or cells transfected with various siRNAs after 24 h were infected with CR at a multiplicity of infection (MOI) of 90 or the medium alone (as a control) or incubated with purified Wnt2b and Wnt5a (@250 ng/ml) for 3 h at 37°C in 5% CO_2_. After 3 h, medium was changed and replaced with fresh medium plus antibiotics to ensure complete absence of live bacteria. Relative levels of activated p65-NF-κB in the nuclear extracts were measured using the TransAm p65 NF-κB Chemi Transcription Factor Assay Kit from Active Motif (Carlsbad, CA) as per manufacturer’s instructions.

### Luciferase Reporter Gene Assay

Transfection experiments were carried out using Lipofectamine 2000 reagent (Invitrogen Life Technologies), according to the manufacturer’s instructions. YAMC cells were maintained as described above. For transient transfections, cells were: i) co-transfected with Tcf-4 reporter plasmid (TOPFlash; TOP) or a mutant Tcf-binding site (FOPFlash; FOP) along with pRL-TK Renilla vector as internal control, or ii) transfected with either 100 nmol/L of scrambled siRNA or siRNAs specific for Wnt2b (sc-155356) and Wnt5a (sc-41113) using 2–8 ml of transfection reagent (sc-29528; Santa Cruz Biotechnology Inc., Santa Cruz, CA). Twenty four hour post-transfection, cells were either uninfected (medium only) or infected with CR (@1∶90 MOI) or incubated with purified Wnt2b and Wnt5a (@250 ng/ml) at 37°C in 5% CO_2_ for 3 hr followed by washing to remove bacteria. At indicated times, cells were processed for measurement of luciferase activity with a luminometer and the values were normalized to the internal control. All transfection experiments were repeated at least three times.

### Knockdown of Wnt/β-catenin Signaling *in vivo* and Western Blotting

For blocking β-catenin *in vivo*, we injected intraperitoneally, β-catenin siRNA incorporated into poly (lactide-co-glycolide) acid nanoparticles (siβ-Cat-NPs) (at 4 µM/Kg. body wt.) every alternate day for 10-days followed by euthanasia at 12-days post-infection [61], [68]. These PLGA-NPs were synthesized using a double emulsion solvent evaporation technique as described [68]. Distal colonic crypts were isolated at various time points as described [34]–[39], [41], [43], [44], [61]–[67] and total crypt cellular or nuclear extracts (30–50 µg protein/lane), were subjected to SDS-PAGE and electrotransferred to nitrocellulose membrane. The membranes were blocked with 5% BSA or 5% nonfat dried milk in Tris-buffered saline (TBS) (20 mM Tris-HCl and 137 mM NaCl, pH 7.5) for 1 h at room temperature (21°C). Immunoantigenicity was detected by incubating the membranes overnight with the appropriate primary antibodies (0.5–1.0 µg/ml in 5% BSA or 5% nonfat dried milk). After washing, membranes were incubated with horseradish peroxidase-conjugated anti-goat, anti-mouse or anti-rabbit secondary antibodies and developed using the ECL detection system (GE) according to the manufacturer’s instructions.

### Histology, Immunofluorescence (IMF) and Immunohistochemistry (IHC)

For histology, tissues were fixed with 10% neutral buffered formalin or in Carnoy’s fixative (60% methanol, 30% chloroform, and 10% acetic acid) prior to paraffin embedding. Paraffin-embedded sections (5 µm) were stained with H&E for gross morphology and the pictures were obtained with an Eclipse E1000 microscope (Nikon). IMF to detect β-catenin in YAMC cells treated as described above or IHCs to detect Ki-67, p65^276^, β-catenin, cyclinD1, CD44, CD133 and Dclk1 were performed on 5-µm-thick paraffin embedded sections prepared from the distal colons of uninfected normal, 3, 5, 7, 9, 12 and 19 days CR-infected or CR-infected and siβ-Cat-NPs treated mice utilizing the HRP labeled polymer conjugated to secondary antibody using Envision+System-HRP (DAB; DakoCytomation, Carpinteria, CA) with microwave accentuation as described previously [34], [36]–[39], [53], [61], [67]. Antibody controls included either omission of the primary antibody or detection of endogenous IgG staining with goat anti-mouse or anti-rabbit IgG (Calbiochem, San Diego, CA). The visualization was carried out using light microscopy. IHC data were graded based on percent of stained cells showing a range of immunoreactivity of: <10% of crypt epithelial cells stained positive, 10 to 30% positivity, 30 to 60% positivity or >60% positivity. A minimum of either ∼200 crypts or ∼2000 cells/field were counted for each experimental group. For statistical analysis of the staining scores, Student’s *t*-test was performed as described below.

### Wound Healing Assay and Electron Microscopy

YAMC cells were transfected with siRNA specific to Wnt2b and Wnt5a, respectively for 24 h. Cells were subsequently treated with control media or with CR (@90∶1 MOI) for 3 h followed by washing to remove bacteria. At 48 h, cells were wounded with a plastic pipette tip. After removing debris and adding fresh media, cell migration was followed for 12–16 h [61]. For electron microscopy, samples of the distal colon from uninfected normal or 9, 12 and 19 days’ CR-infected mice were minced into small cubes and fixed in 4% paraformaldehyde and 2% glutaraldehyde in Cacodylate buffer (0.1 M sodium Cacodylate, pH 7.6) overnight at room temperature and postfixed in 1% Osmium tetroxide for 90 minutes. The fixed tissues were dehydrated through a graded series of ethanols and embedded in epon-araldite resin and maintained for 48 hours at 60°C to polymerize. Ultra-thin (100 nm) sections cut on a Leica UC-6 ultramicrotome were put on glow discharged 300 mesh copper grids and stained with Uranyl Acetate and Sato’s Lead to enhance contrast. Ultra-thin sections were examined with a Hitachi H-7600 electron microscope.

### Statistical Analysis

Experiments were repeated three times with consistent results. Data were expressed as mean values ± standard error. Statistical analyses for all studies were performed using unpaired, two-tailed Student’s *t*-tests and one-way analysis of variance (ANOVA) for multiple group comparisons (GraphPad Prism 5, San Diego, CA). *p*-values <0.05 were considered statistically significant.

## Supporting Information

Figure S1 **Effect of CR infection on NF-κB-p65 phosphorylation in** ***Tlr4^−/−^*** **mice.** Representative photomicrographs of paraffin embedded sections prepared from the distal colons of uninfected normal (N) and days 9–19 post-CR infected *Tlr4^−/−^* mice and stained with H&E for gross morphology (left panel) and for p65 subunit phosphorylated at Ser-276 (p65^276^). Percentages represent percent cells positive for p65^276^. Scale bar: 100 µm; n = 3 independent experiments.
(TIF)

Figure S2 **A. Effect of MEK inhibition on cell migration.** A representative bar graph showing percent migration at 16 h (*, p<0.05 vs. N; **, p<0.05 vs. CR; *^†^, p<0.05 vs. N+MEKi; *♦, p<0.05 vs. CR; n = 3 independent experiments). B. Effect of CR infection on paracellular permeability. FITC-Dextran Assay. Uninfected normal (N) and CR infected C57Bl/6 (C57) or B6.CAST.11M (11M) mice were subjected to gavage with FITC-D, and serum concentrations, shown as fluorescence units, were measured 4 h later (*, p<0.05 vs. control; n = 3 independent experiments).
(TIF)

Figure S3 **Effect of CR infection on recruitment of inflammatory cells.** Representative photomicrographs of paraffin embedded sections prepared from either the distal colons or crypt-denuded lamina propria (CLP) of uninfected normal (N) and days 9–19 post-CR infected C57Bl/6 or B6/CAST.11M mice and stained with antibodies for: CD3+ T cells (upper panel) or F4/80+ macrophages (lower panel). Scale bar: 50 µm; n = 3 independent experiments.
(TIF)

## References

[1] Kemler R (1993) From cadherins to catenins: cytoplasmic protein interactions and regulation of cell adhesion. Trends Genet 9: 317–321. 8236461 10.1016/0168-9525(93)90250-l

[2] Ben-Ze’ev A, Geiger B (1998) Differential molecular interactions of beta-catenin and plakoglobin in adhesion, signaling and cancer. Curr Opin Cell Biol 10: 629–639. 9818174 10.1016/s0955-0674(98)80039-2

[3] Peifer M, Wieschaus E (1990) The segment polarity gene armadillo encodes a functionally modular protein that is the Drosophila homolog of human plakoglobin Cell. 63: 1167–1176. 10.1016/0092-8674(90)90413-9 2261639

[4] McCrea PD, Brieher WM, Gumbiner BM (1993) Induction of a secondary body axis in Xenopus by antibodies to beta-catenin. J Cell Biol 123: 477–484. 8408227 10.1083/jcb.123.2.477 PMC2119835

[5] Funayama N, Fagotto F, McCrea P, Gumbiner BM (1995) Embryonic axis induction by the armadillo repeat domain of beta-catenin: evidence for intracellular signaling. J Cell Biol 128: 959–968. 7876319 10.1083/jcb.128.5.959 PMC2120405

[6] Tanaka K, Kitagawa Y, Kadowaki T (2002) Drosophila segment polarity gene product porcupine stimulates the posttranslational N-glycosylation of wingless in the endoplasmic reticulum. J Biol Chem 277: 12816–12823. 11821428 10.1074/jbc.M200187200

[7] Logan CY, Nusse R (2004) The Wnt signaling pathway in development and disease. Annu Rev Cell Dev Biol 20: 781–810. 15473860 10.1146/annurev.cellbio.20.010403.113126

[8] Torres MA, Yang-Snyder JA, Purcell SM, DeMarais AA, McGrew LL, et al (1996) Activities of the Wnt-1 class of secreted signaling factors are antagonized by the Wnt-5A class and by a dominant negative cadherin in early xenopus development. J Cell Biol 133: 1123–1137. 8655584 10.1083/jcb.133.5.1123 PMC2120849

[9] Topol L, Jiang X, Choi H, Garrett-Beal L, Carolan PJ, et al (2003) Wnt-5a inhibits the canonical Wnt pathway by promoting GSK-3-independent beta-catenin degradation. J Cell Biol 162: 899–908. 12952940 10.1083/jcb.200303158 PMC2172823

[10] Mikels AJ, Nusse R (2006) Wnts as ligands: processing, secretion and reception. Oncogene 25: 7461–8. 17143290 10.1038/sj.onc.1210053

[11] Mikels AJ, Nusse R (2006) Purified Wnt5a protein activates or inhibits beta-catenin-TCF signaling depending on receptor context. PLoSBiol 4: e115. 10.1371/journal.pbio.0040115 PMC1420652 16602827

[12] Baldwin AS (1996) The NF-kappa B and I kappa B proteins: new discoveries and insights. Annu Rev Immunol 14: 649–681. 8717528 10.1146/annurev.immunol.14.1.649

[13] Ghosh S, May MJ, Kopp EB (1998) NF-kappa B and Rel proteins: evolutionarily conserved mediators of immune responses. Annu Rev Immunol 16: 225–260. 9597130 10.1146/annurev.immunol.16.1.225

[14] DiDonato JA, Hayakawa M, Rothwarf DM, Zandi E, Karin M (1997) A cytokine-responsive IkappaB kinase that activates the transcription factor NF-kappaB Nature. 388: 548–554. 10.1038/41493 9252186

[15] Mercurio F, Zhu H, Murray BW, Shevchenko A, Bennett BL, et al (1997) IKK-1 and IKK-2: cytokine-activated IkappaB kinases essential for NF-kappaB activation. Science 278: 860–866. 9346484 10.1126/science.278.5339.860

[16] Regnier CH, Song HY, Gao X, Goeddel DV, Cao Z, et al (1997) Identification and characterization of an IkappaB kinase. Cell 90: 373–383. 9244310 10.1016/s0092-8674(00)80344-x

[17] Woronicz JD, Gao X, Cao Z, Rothe M, Goeddel DV (1997) IkappaB kinase-beta: NF-kappaB activation and complex formation with IkappaB kinase-alpha and NIK. Science 278: 866–869. 9346485 10.1126/science.278.5339.866

[18] Zandi E, Rothwarf DM, Delhase M, Hayakawa M, Karin M (1997) The IkappaB kinase complex (IKK) contains two kinase subunits, IKKalpha and IKKbeta, necessary for IkappaB phosphorylation and NF-kappaB activation. Cell 91: 243–252. 9346241 10.1016/s0092-8674(00)80406-7

[19] Hoeflich KP, Luo J, Rubie EA, Tsao MS, Jin O, et al (2000) Requirement for glycogen synthase kinase-3β in cell survival and NF-κB activation. Nature 406: 86–90. 10894547 10.1038/35017574

[20] Veeman MT, Axelrod JD, Moon RT (2003) A second canon. Functions and mechanisms of beta-catenin-independent Wnt signaling. Dev Cell 5: 367–377. 12967557 10.1016/s1534-5807(03)00266-1

[21] Witze ES, Litman ES, Argast GM, Moon RT, Ahn NG (2008) Wnt5a control of cell polarity and directional movement by polarized redistribution of adhesion receptors. Science 320: 365–369. 18420933 10.1126/science.1151250 PMC3229220

[22] Weeraratna AT, Jiang Y, Hostetter G, Rosenblatt K, Duray P, et al (2002) Wnt5a signaling directly affects cell motility and invasion of metastatic melanoma. Cancer Cell 1: 279–288. 12086864 10.1016/s1535-6108(02)00045-4

[23] Kurayoshi M, Oue N, Yamamoto H, Kishida M, Inoue A, et al (2006) Expression of Wnt-5a is correlated with aggressiveness of gastric cancer by stimulating cell migration and invasion. Cancer Res 66: 10439–10448. 17079465 10.1158/0008-5472.CAN-06-2359

[24] Sen M, Ghosh G (2008) Transcriptional outcome of Wnt-Frizzled signal transduction in inflammation: evolving concepts. J Immunol 181: 4441–4445. 18802045 10.4049/jimmunol.181.7.4441

[25] George SJ (2008) Wnt pathway: a new role in regulation of inflammation. Arterioscler Thromb Vasc Biol 28: 400–402. 18296599 10.1161/ATVBAHA.107.160952

[26] Blumenthal A, Ehlers S, Lauber J, Buer J, Lange C, et al (2006) The Wingless homolog WNT5A and its receptor Frizzled-5 regulate inflammatory responses of human mononuclear cells induced by microbial stimulation. Blood 108: 965–973. 16601243 10.1182/blood-2005-12-5046

[27] Sen M, Lauterbach K, El-Gabalawy H, Firestein GS, Corr M, et al (2000) Expression and function of wingless and frizzled homologs in rheumatoid arthritis. Proc Natl Acad Sci USA 97: 2791–2796. 10688908 10.1073/pnas.050574297 PMC16008

[28] Sodhi CP, Shi XH, Richardson WM, Grant ZS, Shapiro RA, et al (2010) Toll-like receptor-4 inhibits enterocyte proliferation via impaired beta-catenin signaling in necrotizing enterocolitis. Gastroenterology 138: 185–96. 19786028 10.1053/j.gastro.2009.09.045 PMC2813409

[29] Gribar SC, Sodhi CP, Richardson WM, Anand RJ, Gittes GK, et al (2009) Reciprocal expression and signaling of TLR4 and TLR9 in the pathogenesis and treatment of necrotizing enterocolitis. J Immunol 182: 636–646. 19109197 10.4049/jimmunol.182.1.636 PMC3761063

[30] Cetin S, Ford HR, Sysko LR, Agarwal C, Wang J, et al (2004) Endotoxin inhibits intestinal epithelial restitution through activation of Rho-GTPase and increased focal adhesions. J Biol Chem 279: 24592–24600. 15169791 10.1074/jbc.M313620200

[31] Mundy R, MacDonald TT, Dougan G, Frankel G, Wiles S (2005) *Citrobacter rodentium* of mice and man. Cell Microbiol 7: 1697–1706. 16309456 10.1111/j.1462-5822.2005.00625.x

[32] Borenshtein D, McBee ME, Schauer DB (2008) Utility of the *Citrobacter rodentium* infection model in laboratory mice. Curr Opin Gastroenterol 24: 32–37. 18043230 10.1097/MOG.0b013e3282f2b0fb

[33] Barthold SW, Coleman GL, Jacoby RO, Livestone EM, Jonas AM (1978) Transmissible murine colonic hyperplasia. Vet Pathol 15: 223–236. 664189 10.1177/030098587801500209

[34] Wang Y, Kourouma F, Guang-Sheng X, Umar S (2006) *Citrobacter rodentium*-induced NF-κB activation in hyperproliferating colonic epithelia: role of p65 (Ser^536^) phosphorylation. British J Pharmacol 148: 814–824. 10.1038/sj.bjp.0706784 PMC1617077 16751795

[35] Umar S, Wang Y, Morris AP, Sellin JH (2007) Dual alterations in casein kinase 1ε and GSK-3β modulate β-catenin stability in hyperproliferating colonic epithelia. Am. J. Physiol Gastrointest Liver Physiol 292: G599–G607. 10.1152/ajpgi.00343.2006 17053159

[36] Sellin JH, Wang Y, Singh P, Umar S (2009) β-Catenin stabilization imparts crypt progenitor phenotype to hyperproliferating colonic epithelia. Exp Cell Res 315: 97–109. 18996369 10.1016/j.yexcr.2008.10.019 PMC2868370

[37] Ahmed I, Chandrakesan P, Tawfik O, Xia L, Anant S, et al (2012) Critical roles of Notch and Wnt/β-catenin pathways in the regulation of hyperplasia and/or colitis in response to bacterial infection. Infect Immun 80: 3107–3121. 22710872 10.1128/IAI.00236-12 PMC3418747

[38] Ahmed I, Roy B, Chandrakesan P, Venugopal A, Xia L, et al (2013) Evidence of functional cross talk between the Notch and NF-κB pathways in non-neoplastic hyperproliferating colonic epithelium. Am J Physiol Gastrointest Liver Physiol 304: G356–370. 23203159 10.1152/ajpgi.00372.2012 PMC3566617

[39] Chandrakesan P, Ahmed I, Chinthalapally A, Singh P, Awasthi S, et al (2012) Distinct compartmentalization of Nuclear Factor-κB activity in the crypt and crypt-denuded lamina propria precede and accompany hyperplasia and/or colitis following bacterial infection. Infect Immun 80: 753–767. 22144489 10.1128/IAI.06101-11 PMC3264290

[40] Khan MA, Ma C, Knodler LA, Valdez Y, Rosenberger CM, et al (2006) Toll-like receptor 4 contributes to colitis development but not to host defense during Citrobacter rodentium infection in mice. Infect Immun 74: 2522–2536. 16622187 10.1128/IAI.74.5.2522-2536.2006 PMC1459750

[41] Chandrakesan P, Ahmed I, Anwar T, Wang Y, Sarkar S et al.. (2010) Novel changes in NF-κB activity during progression and regression phases of hyperplasia: Role of ERK1/2 and p38. J Biol Chem. 10.1074/jbc.M110.129353 PMC2963366 20710027

[42] Fukata M, Michelsen KS, Eri R, Thomas LS, Hu B, et al (2005) Toll-like receptor-4 is required for intestinal response to epithelial injury and limiting bacterial translocation in a murine model of acute colitis. Am J Physiol Gastrointest Liver Physiol 288: G1055–1065. 15826931 10.1152/ajpgi.00328.2004

[43] Umar S, Wang Y, Sellin JH (2005) Epithelial proliferation induces novel changes in APC expression Oncogene. 24: 6709–6718. 10.1038/sj.onc.1208820 16007167

[44] Umar S, Sellin JH, Morris AP (2000) Increased Nuclear Translocation of Catalytically Active PKC-ζ During Mouse Colonocyte Hyperproliferation. Am J Physiol Gastrointest Liver Physiol 279: G223–G237. 10898766 10.1152/ajpgi.2000.279.1.G223

[45] Katoh M (2005) WNT2B: comparative integromics and clinical applications. Int J Mol Med 16: 1103–1108. 16273293

[46] Moayeri M, Martinez NW, Wiggins J, Young HA, Leppla SH (2004) Mouse susceptibility to anthrax lethal toxin is influenced by genetic factors in addition to those controlling macrophage sensitivity. Infect Immun 72: 4439–4447. 15271901 10.1128/IAI.72.8.4439-4447.2004 PMC470648

[47] Terra JK, France B, Cote CK, Jenkins A, Bozue JA, et al (2011) Allelic variation on murine chromosome 11 modifies host inflammatory responses and resistance to Bacillus anthracis. PLoS Pathog 7: e1002469. 22241984 10.1371/journal.ppat.1002469 PMC3248472

[48] Kawai T, Akira S (2010) The role of pattern-recognition receptors in innate immunity: update on Toll-like receptors. Nature Immunology 11: 3730384. 10.1038/ni.1863 20404851

[49] Santaolalla R, Sussman DA, Ruiz JR, Davies JM, Pastorini C, et al (2013) TLR4 activates the β-catenin pathway to cause intestinal neoplasia. PLoS One 8: e63298. 23691015 10.1371/journal.pone.0063298 PMC3653932

[50] Fukata M, Chen A, Vamadevan AS, Cohen J, Breglio K, et al (2007) Toll-like receptor-4 promotes the development of colitis-associated colorectal tumors. Gastroenterology 133: 1869–1881. 18054559 10.1053/j.gastro.2007.09.008 PMC2180834

[51] Fukata M, Shang L, Santaolalla R, Sotolongo J, Pastorini C, et al (2011) Constitutive activation of epithelial TLR4 augments inflammatory responses to mucosal injury and drives colitis-associated tumorigenesis. Inflamm Bowel Dis 17: 1464–1473. 21674704 10.1002/ibd.21527 PMC3117047

[52] Vlantis K, Wullaert A, Sasaki Y, Schmidt-Supprian M, Rajewsky K, et al (2011) Constitutive IKK2 activation in intestinal epithelial cells induces intestinal tumors in mice. J Clin Invest 121: 2781–2793. 21701067 10.1172/JCI45349 PMC3223831

[53] Umar S, Sarkar S, Wang Y, Singh P (2009) Functional cross-talk between β-catenin and NF-κB signaling pathways in colonic crypts of mice in response to Progastrin. J Biol Chem 284: 22274–22284. 19497850 10.1074/jbc.M109.020941 PMC2755951

[54] Du Q, Geller DA (2010) Cross-regulation between Wnt and NF-κB signaling pathways. For Immunopathol Dis Therap 1: 155–181. 10.1615/ForumImmunDisTher.v1.i3 PMC3114374 21686046

[55] Katoh M, Katoh M (2009) Transcriptional regulation of WNT2B based on the balance of Hedgehog, Notch, BMP and WNT signals. Int J Oncol 34: 1411–1415. 19360354

[56] Tanaka A, Leung PS, Kenny TP, Au-Young J, Prindiville T, et al (2001) Genomic analysis of differentially expressed genes in liver and biliary epithelial cells of patients with primary biliary cirrhosis. J Autoimmun 17: 89–98. 11488641 10.1006/jaut.2001.0522

[57] You J, Nguyen AV, Albers CG, Lin F, Holcombe RF (2008) Wnt pathway-related gene expression in inflammatory bowel disease. Dig Dis Sci 53: 1013–1019. 17939044 10.1007/s10620-007-9973-3

[58] Goss AM, Tian Y, Tsukiyama T, Cohen ED, Zhou D, et al (2009) Wnt2/2b and beta-catenin signaling are necessary and sufficient to specify lung progenitors in the foregut. Dev Cell 17: 290–298. 19686689 10.1016/j.devcel.2009.06.005 PMC2763331

[59] Iakoubova OA, Olsson CL, Dains KM, Ross DA, Andalibi A, et al (2001) Genome-tagged mice (GTM): two sets of genome-wide congenic strains. Genomics 74: 89–104. 11374905 10.1006/geno.2000.6497

[60] Davis RC, Jin A, Rosales M, Yu S, Xia X, et al (2007) A genome-wide set of congenic mouse strains derived from CAST/Ei on a C57BL/6 background. Genomics 90: 306–313. 17600671 10.1016/j.ygeno.2007.05.009

[61] Chandrakesan P, Roy BC, Jakkula LUMR, Ahmed I, Ramamoorthy P, et al. (2013) Utility of a bacterial infection model to study Epithelial-Mesenchymal Transition (EMT), Mesenchymal-Epithelial Transition (MET) or tumorigenesis. Oncogene doi: 10.1038/onc.2013.210 10.1038/onc.2013.210 PMC3883801 23752178

[62] Umar S, Morris AP, Kourouma F, Sellin JH (2003) Dietary pectin and calcium inhibit colonic proliferation *in vivo* by differing mechanisms. Cell Prolif 36: 361–375. 14710853 10.1046/j.1365-2184.2003.00291.x PMC6496283

[63] Umar S, Scott J, Sellin JH, Dubinsky WP, Morris AP (2000) Murine colonic mucosa hyperproliferation: 1) Elevated CFTR expression and enhanced cAMP-dependent Cl^−^ secretion. Am J Physiol Gastrointest Liver Physiol 278: G753–G764. 10801268 10.1152/ajpgi.2000.278.5.G753

[64] Umar S, Scott J, Sellin JH, Morris AP (2000) Murine colonic mucosa hyperproliferation: 2) PKC-β activation and cPKC mediated cellular CFTR over-expression. Am J Physiol Gastrointest Liver Physiol 278: G765–G774. 10801269 10.1152/ajpgi.2000.278.5.G765

[65] Sellin J, Umar S, Xio J, Morris AP (2001) Increased homotypic β-catenin expression and nuclear translocation accompany cellular hyper-proliferation *in vivo* . Cancer Res 61: 2899–2906. 11306465

[66] Peleg S, Sellin JH, Wang Y, Freeman MR, Umar S (2010) Suppression of aberrant transient receptor potential cation channel, subfamily V, member 6 expression in hyperproliferative colonic crypts by dietary calcium. Am. J. Physiol. 10.1152/ajpgi.00193.2010 PMC2950683 20508153

[67] Umar S, Sarkar S, Cowey S, Singh P (2008) Activation of NF-κB is required for mediating proliferative and anti-apoptotic effects of progastrin on proximal colonic crypts of mice, *in vivo.* . Oncogene 27: 5599–5611. 18521082 10.1038/onc.2008.169 PMC2891442

[68] Cun D, Jensen DK, Maltesen MJ, Bunker M, Whiteside P, et al (2011) High loading efficiency and sustained release of siRNA encapsulated in PLGA nanoparticles: quality by design optimization and characterization. Eur J Pharm Biopharm 77: 26–235. 21093589 10.1016/j.ejpb.2010.11.008

