# Supplementary figures and images for: Differential Effects of β-catenin and NF-κB Interplay in the Regulation of Cell Proliferation, Inflammation and Tumorigenesis in Response to Bacterial Infection
Source: PLoS One. 2013 Nov 21;8(11):e79432. doi: 10.1371/journal.pone.0079432 (PMC3836902; doi:10.1371/journal.pone.0079432)

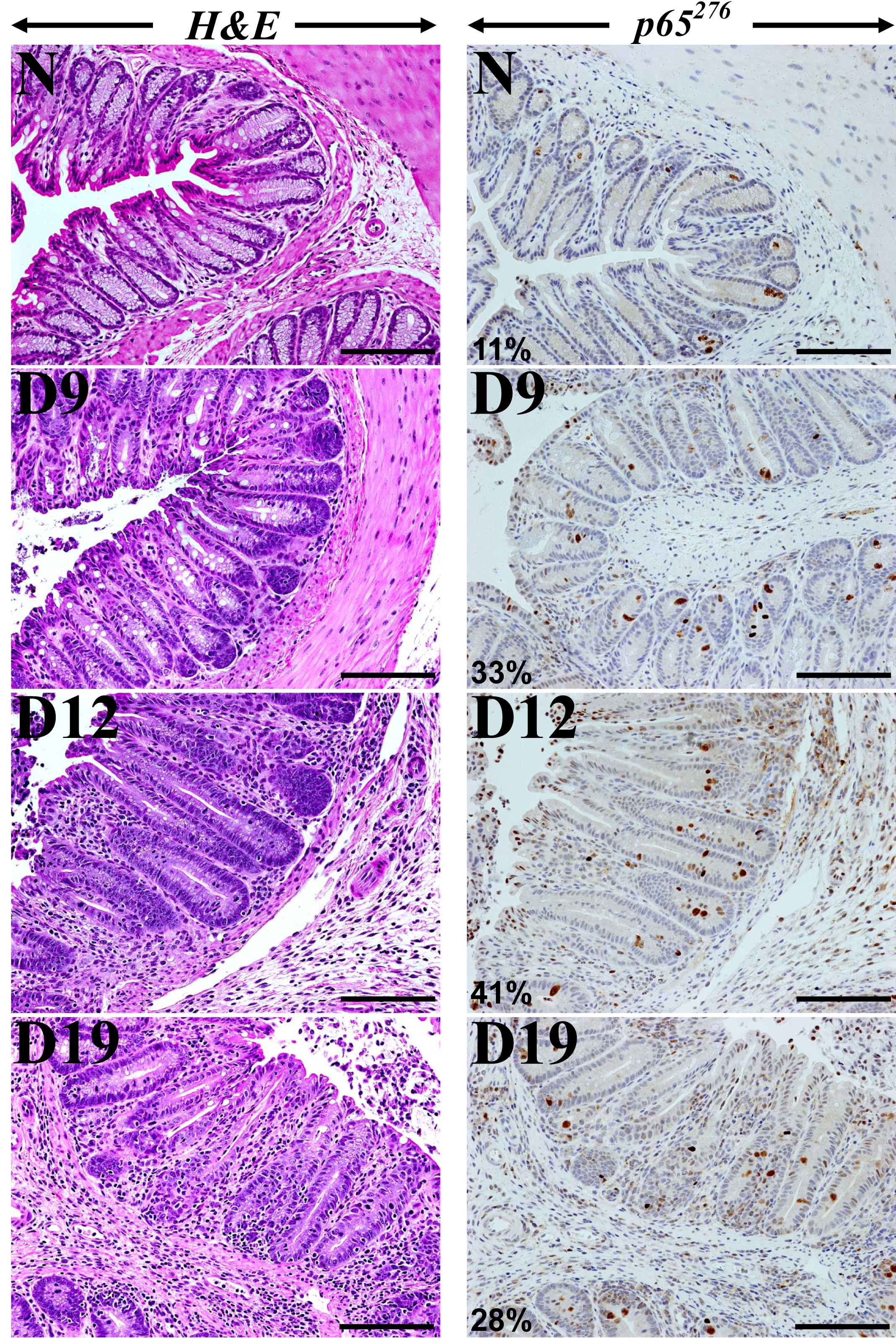

Supplement: Figure S1 — Effect of CR infection on NF-κB-p65 phosphorylation in Tlr4−/− mice. Representative photomicrographs of paraffin embedded sections prepared from the distal colons of uninfected normal (N) and days 9–19 post-CR infected Tlr4−/− mice and stained with H&E for gross morphology (left panel) and for p65 subunit phosphorylated at Ser-276 (p65276). Percentages represent percent cells positive for p65276. Scale bar: 100 µm; n = 3 independent experiments. (TIF) [file pone.0079432.s001.tif]

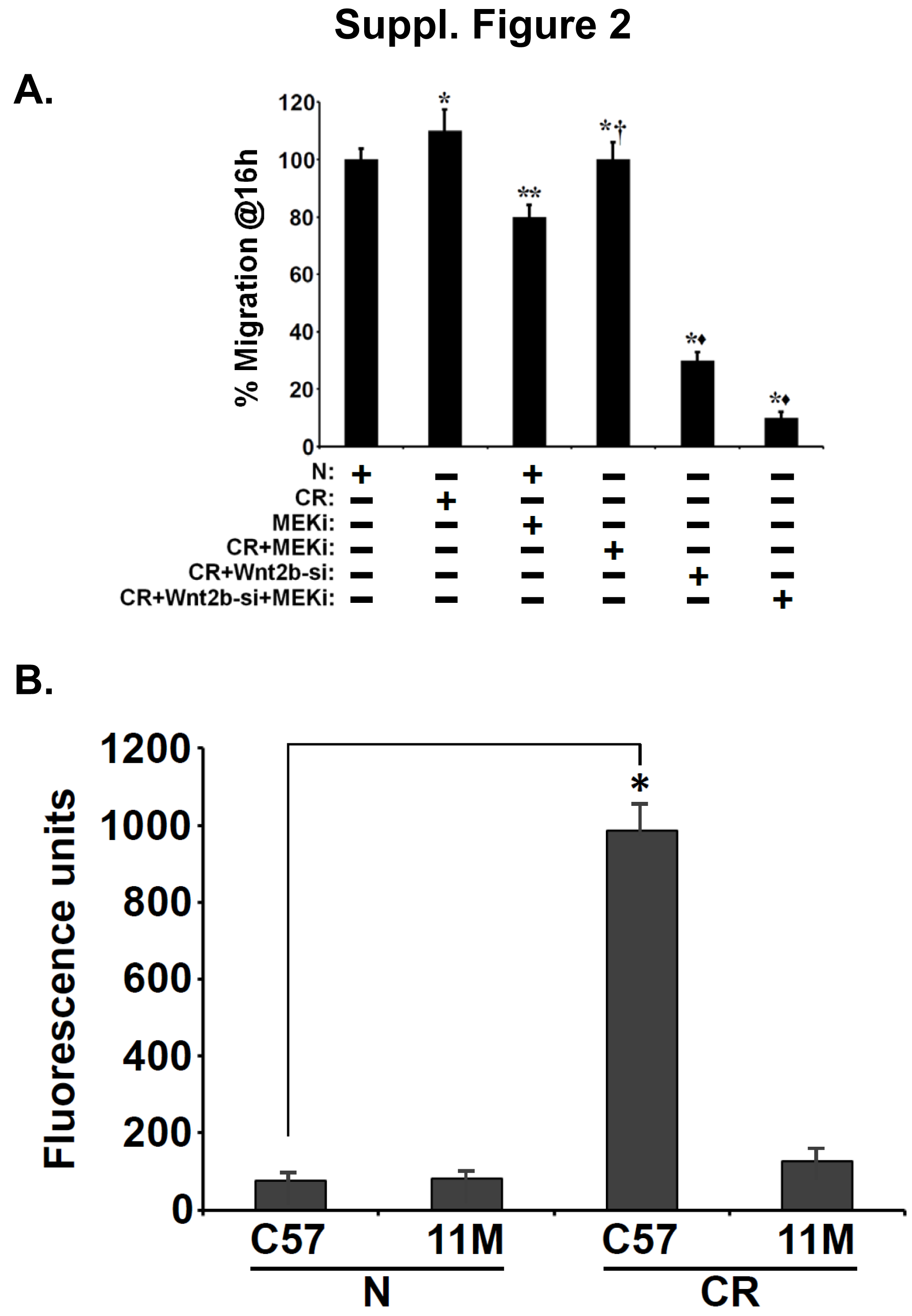

Supplement: Figure S2 — A. Effect of MEK inhibition on cell migration. A representative bar graph showing percent migration at 16 h (*, p<0.05 vs. N; **, p<0.05 vs. CR; *†, p<0.05 vs. N+MEKi; *♦, p<0.05 vs. CR; n = 3 independent experiments). B. Effect of CR infection on paracellular permeability. FITC-Dextran Assay. Uninfected normal (N) and CR infected C57Bl/6 (C57) or B6.CAST.11M (11M) mice were subjected to gavage with FITC-D, and serum concentrations, shown as fluorescence units, were measured 4 h later (*, p<0.05 vs. control; n = 3 independent experiments). (TIF) [file pone.0079432.s002.tif]

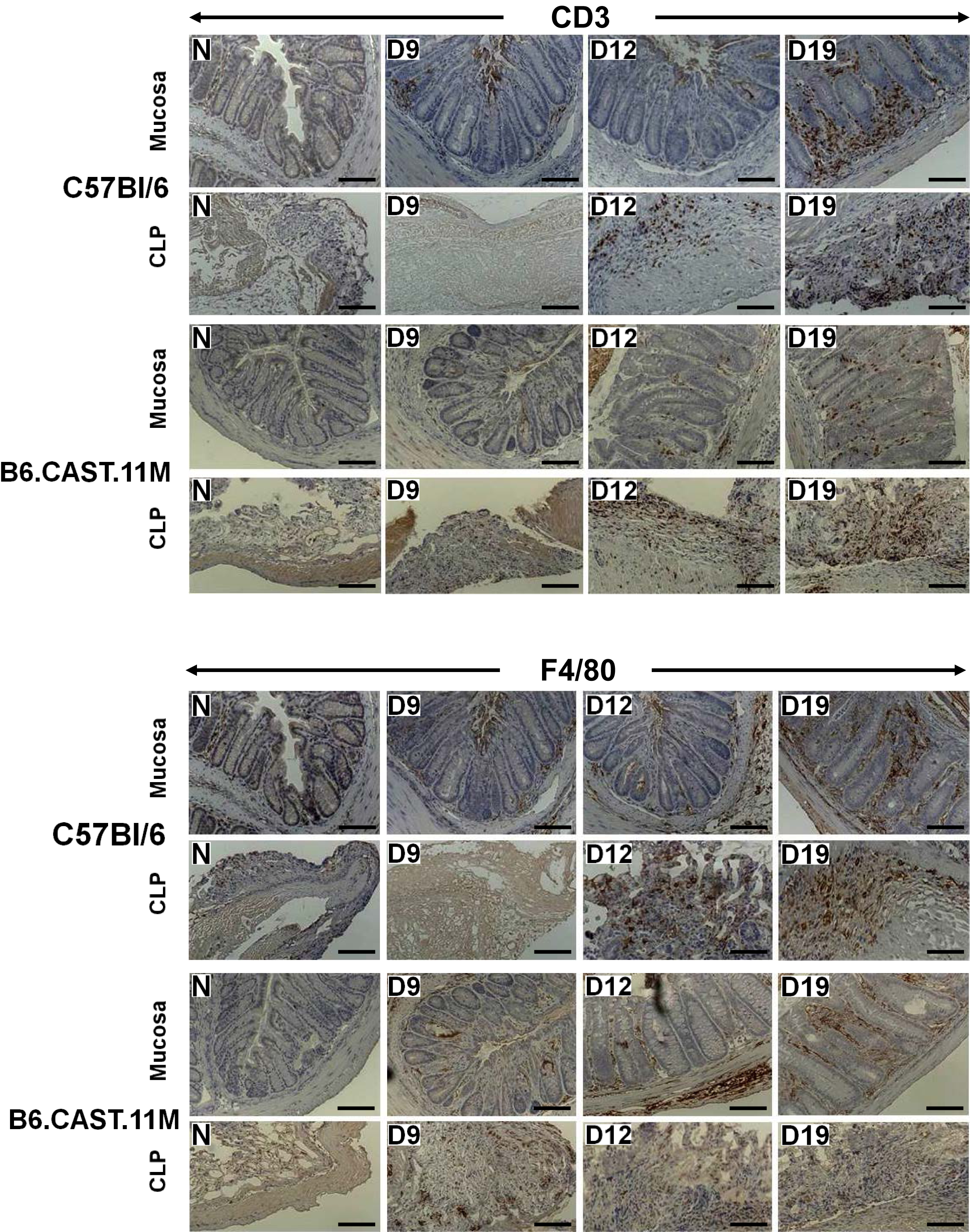

Supplement: Figure S3 — Effect of CR infection on recruitment of inflammatory cells. Representative photomicrographs of paraffin embedded sections prepared from either the distal colons or crypt-denuded lamina propria (CLP) of uninfected normal (N) and days 9–19 post-CR infected C57Bl/6 or B6/CAST.11M mice and stained with antibodies for: CD3+ T cells (upper panel) or F4/80+ macrophages (lower panel). Scale bar: 50 µm; n = 3 independent experiments. (TIF) [file pone.0079432.s003.tif]
